# Biochemical Markers of Calcium and Bone Metabolism during and after Lactation in Ugandan Women with HIV on Universal Maternal Antiretroviral Therapy

**DOI:** 10.1002/jbmr.4866

**Published:** 2023-07-24

**Authors:** Florence Nabwire, Matthew M. Hamill, Mary Glenn Fowler, Josaphat Byamugisha, Adeodata Kekitiinwa, Ann Prentice

**Affiliations:** ^1^ MRC Epidemiology Unit University of Cambridge Cambridge UK; ^2^ MRC Nutrition and Bone Health Research Group Cambridge UK; ^3^ Formely based at the MRC Elsie Widdowson Laboratory Cambridge UK; ^4^ Johns Hopkins University School of Medicine Baltimore MD USA; ^5^ Makerere University Kampala Uganda; ^6^ Baylor College of Medicine Children's Foundation – Uganda (Baylor‐Uganda) Kampala Uganda

**Keywords:** AFRICAN WOMEN, BIOCHEMICAL MARKERS, HIV, PREGNANCY AND LACTATION, TENOFOVIR‐BASED ANTIRETROVIRAL THERAPY (ART)

## Abstract

We reported accentuated lactational decreases in areal bone mineral density and only partial skeletal recovery after lactation in Ugandan women with HIV (WWH) initiated on tenofovir disoproxil fumarate‐based antiretroviral therapy (TDF‐based ART) during pregnancy compared to women without HIV (REF). WWH also had higher breast milk calcium in the first months of lactation. To investigate the mechanisms, we measured bone turnover markers (bone resorption: C‐terminal telopeptide [CTX]; bone formation: procollagen type 1 N‐terminal propeptide [P1NP], bone‐specific and total alkaline phosphatase [BALP, TALP]), hormones (parathyroid hormone [PTH], intact fibroblast growth factor 23 [FGF23], 1,25‐dihydroxyvitamin D [1,25(OH)_2_D]), vitamin D status (25‐hydroxyvitamin D [25OHD]), and indices of mineral metabolism and renal function. Blood and urine samples collected at 36 weeks of gestation, 14 and 26 weeks of lactation, and 3–6 months after lactation were analyzed. Mean 25OHD was >50 nmol/L throughout. Both groups experienced similar biochemical changes during pregnancy and lactation to women in other settings, but within these patterns, the two groups differed significantly. Notably, WWH had higher PTH (+31%) and lower 1,25(OH)_2_D (−9%) and TmP/GFR (−9%) throughout, lower P1NP (−27%) and plasma phosphate (−10%) in pregnancy, higher CTX (+15%) and BALP (+19%), and lower eGFR (−4%) during and after lactation. P1NP/CTX ratio was lower in WWH than REF in pregnancy (−21%), less so in lactation (−15%), and similar after lactation. Additionally, WWH had lower plasma calcium (−5%), lower FGF23 (−16%) and fasting urinary calcium (−34%) at one or both lactation timepoints, and higher fasting urinary phosphate (+22%) at 26 weeks of lactation and after lactation. These differences resemble reported TDF effects, especially raised PTH, increased bone resorption, decreased bone formation, and decreased renal function, and may explain the observed differences in bone mineral density and breast milk calcium. Further studies are needed to determine whether HIV and TDF‐based ART have long‐term consequences for maternal bone health and offspring growth. © 2023 The Authors. *Journal of Bone and Mineral Research* published by Wiley Periodicals LLC on behalf of American Society for Bone and Mineral Research (ASBMR).

## Introduction

The World Health Organization (WHO) recommends universal maternal antiretroviral therapy (ART) in pregnant and lactating women living with HIV (WWH) for the prevention of mother‐to‐child HIV transmission and breastfeeding for 12–24 months for optimal HIV‐free offspring survival in resource‐limited settings.^(^ ^1^, ^2^ ^)^ Many studies have reported declines in areal bone mineral density (aBMD) following ART initiation in those with^(^ ^3^, ^4^, ^5^ ^)^ and without HIV where ART is used as prophylaxis.^(^ ^6^ ^)^ Tenofovir disoproxil fumarate (TDF)‐containing ART, the current first‐line regimen for adults in Sub‐Saharan Africa (SSA), including pregnant and lactating women, is associated with greater bone loss and fracture risk than regimens without TDF.^(^ ^7^, ^8^ ^)^ Some of the mechanisms proposed for TDF‐associated bone loss include increased bone turnover, delayed bone formation, elevated parathyroid hormone (PTH), low 25‐hydroxyvitamin D (25OHD) and altered vitamin D metabolism, and renal toxicity causing phosphate wasting.^(^ ^8^, ^9^, ^10^ ^)^ However, few studies have investigated changes in maternal bone and mineral metabolism following initiation of ART (with or without TDF) in pregnant and lactating WWH.^(^ ^11^, ^12^ ^)^

Maternal calcium requirements increase during pregnancy and lactation to supply calcium for offspring bone mineral accretion. The mean calcium accretion rate in the fetus increases from 50 mg/day at 20 weeks to about 300–350 mg/day at 35 weeks of gestation, and 200–400 mg of calcium is secreted into breast milk at peak lactation.^(^ ^13^, ^14^ ^)^ These increased calcium requirements are met through physiological adaptations, which may involve increased intestinal calcium absorption efficiency, enhanced renal calcium retention, and mobilization of skeletal calcium, which reduces maternal aBMD especially during lactation.^(^ ^13^, ^14^ ^)^ To date, most,^(^ ^13^, ^14^ ^)^ but not all,^(^ ^15^, ^16^ ^)^ studies suggest that maternal aBMD recovers in later months of pregnancy and after lactation in apparently healthy mothers, even after successive periods of prolonged lactation in African women accustomed to a low‐calcium diet.^(^ ^17^ ^)^ Biochemical data have shown altered concentrations of bone turnover markers during and after lactation consistent with changes in aBMD.^(^ ^13^, ^14^ ^)^ Differences in the timing of these changes between bone resorption and bone formation are thought to account for bone mineral mobilization in early lactation, when resorption exceeds formation, and recovery of bone mineral in late lactation and after lactation, when formation exceeds resorption.^(^ ^13^ ^)^ However, it is unknown whether this is the case for WWH on ART and whether skeletal recovery is achieved after lactation in these women.

We recently reported, for the first time, greater decreases in aBMD during lactation and only partial skeletal recovery at 3–6 months after lactation in a cohort of Ugandan WWH initiated on TDF‐containing ART in pregnancy compared to counterparts not living with HIV (REF).^(^ ^11^ ^)^ We also reported higher breast milk calcium concentrations during the first months of lactation in WWH than REF from the same study cohort.^(^ ^18^ ^)^ The purpose of the study reported here was to chart changes in biochemical markers of bone and calcium metabolism from late pregnancy to 3–6 months after lactation in these women in order to investigate the metabolic effects of HIV/ART and identify potential mechanisms for the observed patterns of change in aBMD between Ugandan WWH and REF.

## Participants and Methods

### Study details

The biological samples in this study were collected during an investigation of maternal ART and bone health in Kampala, Uganda, known locally as the Gumba (Bone) Study.^(^ ^11^, ^18^ ^)^ This was a prospective, longitudinal study of changes in aBMD and biochemical markers of calcium metabolism and bone turnover during pregnancy, lactation, and after lactation to compare previously ART‐naïve WWH initiated on ART in pregnancy with REF counterparts who were without HIV. Ethics and protocol approvals were obtained from the institutional review boards of the Joint Clinical Research Centre and Mulago Hospital, Kampala, Uganda, and from the Uganda National Council for Science and Technology. Between January 2015 and September 2017, 100 WWH and 100 REF pregnant women with singleton pregnancies were recruited at the midwife‐led (low‐risk) antenatal clinic at Mulago National Referral Hospital, Kampala, Uganda. After providing written informed consent, they were invited for measurements at 36 weeks of gestation (P36), postpartum at 2 (L2), 14 (L14), 26 (L26), and 52 (L52) weeks of lactation, and at least 3 months after stopping breastfeeding when neither pregnant nor lactating (NPNL). Blood and urine samples were collected at P36, L14, L26, and NPNL.

Inclusion in the Gumba Study was based on the following criteria: less than 36 weeks of gestation with a documented rapid HIV test from Mulago Hospital during the index pregnancy using the nationally approved sequential rapid HIV testing algorithm, aged 18.0–39.9 years old with no known medical complications, planning to breastfeed for at least 6 months, and not planning to move from the Kampala catchment area in the following year. Exclusions included a nonsingleton pregnancy, an HIV diagnosis or ART prior to the index pregnancy, a pregnancy classified as high‐risk (hypertension, preeclampsia/eclampsia), diagnosis of bone disease, diabetes mellitus, gestational diabetes, tuberculosis, hepatitis, proteinuria, or renal disease. Later exclusions included delivery <37 weeks of gestation, stillbirth, neonatal death, stopping breastfeeding before 14 weeks postpartum or subsequent pregnancy. The sample size of the Gumba Study was based on a mean percentage difference between WWH and REF in change in lumbar spine aBMD during early lactation of 2% (SD 4%) at 80% power and a type 1 error of 0.05 (two‐tailed).^(^ ^11^ ^)^ Differences between groups in breast‐milk composition and biochemical markers were secondary outcomes.

The participants in the Gumba Study were at weeks 24–32 of gestation at enrollment. All WWH women had been initiated onto ART during the index pregnancy at a mean (SD) 10.8 (SD 5.0) weeks prior to the P36 measurements. The ART regimen was first‐line “option B+” (since subsumed under universal maternal ART guidelines), consisting of TDF‐lamivudine (3TC)‐efavirenz (EFV). ART was initiated following a positive HIV rapid test at an antenatal visit prior to enrollment; 61.3% had CD_4_ counts above 350 cells/mm^3^ at ART initiation, and 95% reported excellent adherence (>90% based on pill count method). Women in the REF group were ART‐naïve and had a reported negative HIV test result. No participant reported use of calcium‐containing supplements, and there was no difference between the groups in the inclusion of calcium‐rich foods in their usual diet (such as milk or small fish eaten with bones), except for a greater frequency of use of cooked dry beans and peas by WWH. Maternal characteristics, aBMD, body composition, anthropometry, and breast milk composition of the full cohort are described elsewhere.^(^ ^11^, ^18^ ^)^

### Biological samples: Collection, processing, and laboratory analysis

The 2‐h fasting protocol by Nordin et al.^(^ ^19^ ^)^ was followed to collect and process blood and urine samples, as detailed in Supplement 1 in Data S1. Processed blood and urine samples were stored at −80°C in Uganda and later airfreighted on dry ice, in batches, for storage and laboratory analysis at the Medical Research Council (MRC) Elsie Widdowson Laboratory and the MRC Epidemiology Unit in Cambridge, UK. Full details of the collection processes, assays, and formulas used are given in Supplement 1 in Data S1.

Blood and urine samples were analyzed for bone turnover markers (bone resorption marker: C‐terminal telopeptide [CTX]; bone formation markers: procollagen type 1 N‐terminal propeptide [P1NP], bone‐specific and total alkaline phosphatase [BALP, TALP]), hormones involved in calcium and phosphorus metabolism (intact parathyroid hormone [PTH], intact fibroblast growth factor‐23 [FGF23], 1,25‐dihydroxyvitamin D [1,25(OH)_2_D]), vitamin D status (25‐hydroxyvitamin D [25OHD]), and indices of mineral metabolism (plasma calcium, phosphate, magnesium, albumin, and creatinine concentrations [pCa, pP, pMg, pAlb, pCr, respectively] and 2 h fasting urinary calcium, phosphate, magnesium, and creatinine concentrations [uCa, uP, uMg, uCr respectively]). The ratio of P1NP/CTX was used as an index of the balance between bone formation and bone resorption.^(^ ^20^ ^)^

The Payne equation was used to normalize pCa for pAlb to provide a proxy for circulating ionized calcium (pCa_corr_).^(^ ^21^ ^)^ Urinary mineral concentrations from the 2 h fasting collections were expressed relative to uCr concentration (uCa/Cr, uP/Cr, uMg/Cr) and used to calculate renal tubular maximum reabsorption of Ca and P per unit volume of glomerular filtration (TmCa/GFR, TmP/GFR). Estimated glomerular filtration rate (eGFR) based on plasma creatinine (pCr) was calculated using the Chronic Kidney Disease – Epidemiology Collaboration (CKD‐EPI) equation for females,^(^ ^22^ ^)^ without the correction for black ethnicity, consistent with previous studies in African populations.^(^ ^4^, ^23^ ^)^

### Statistical methods

Data were analyzed using DataDesk 6.3.1 software (Data Description Inc, Ithaca, NY, USA). Descriptive statistics for participant characteristics are presented as mean (SD) for normally distributed data, medians (25th percentile, 75th percentile) for skewed distributions, and percentages (%) for proportions. Prevalence estimates of low vitamin D status were calculated using 25 nmol/L^(^ ^24^ ^)^ and 50 nmol/L^(^ ^25^ ^)^ as thresholds.

Statistical models were established using all biochemical variables transformed into natural logarithms (log_e_), and all biochemical data are presented as geometric means (25th percentile, 75th percentile) calculated by back‐transformation of log_e_ values. This conversion enabled the changes within group and differences between groups to be expressed as sympercents ([difference/mean] × 100%),^(^ ^26^ ^)^ thereby facilitating comparisons of relative size effects between the biochemical markers. The conversion also normalized positively skewed distributions. A sensitivity analysis was conducted by repeating the models with data restricted to participants that provided samples at all four timepoints (rectangular dataset). A two‐tailed *p* value of ≤0.05 was considered significant for all tests.

Within‐individual changes over time in each group, differences between groups at each timepoint, and differences between groups in change over time were investigated using repeated‐measures ANOVA in four‐time‐point hierarchical/nested models (P36‐L14‐L26‐NPNL). These were constructed for each variable with an individual identifier (nested by group), timepoint, group, and a group × timepoint interaction term. Scheffé post hoc tests were used to account for multiple testing and to provide estimates (expressed as means [95% confidence interval, CI]) of the size and significance of within‐group changes between timepoints (%∆) and of between‐group differences at each timepoint (%Diff). Where the interaction term was not significant, it was removed to give an estimate of the overall difference between groups.

As the four‐timepoint models could only test for the significance of differences between groups in the overall patterns of change between P36 and NPNL (as given by the *p* value for group × timepoint interaction), separate three‐timepoint hierarchical models were constructed to compare patterns of change in biochemical markers from late pregnancy to lactation (P36‐L14‐L26) and from lactation to NPNL (L14‐L26‐NPNL). Additionally, two‐timepoint hierarchical models were constructed pairwise to test for differences between groups in change between successive timepoints (P36‐L14, L14‐L26, L26‐NPNL) and between pregnancy and NPNL (P36‐NPNL).

The influence of potential confounders on differences between WWH and REF at each timepoint was explored for each analyte using multivariate regression to enable those variables that did not change over time to be included. The following variables were considered: at P36, maternal age (years), height (log_e_ transformed), body weight (log_e_ transformed), primigravidity 1/0, and previous use of depot medroxyprogesterone acetate (DMPA) 1/0; at L14 and L26 as for P36 plus current exclusive breastfeeding 1/0; menses resumed 1/0, infant sex (M/F, 1/2), and current use of DMPA 1/0; at NPNL as for P36 plus infant sex, current use of DMPA 1/0 and duration of breastfeeding (months). Additionally, the influence of these variables on change between timepoints was considered in repeated‐measures ANOVA and ANCOVA models. Adjustment for potential confounders in both these sets of models was performed by backward elimination of nonsignificant variables, the least significant first, to produce parsimonious models.

The possible effect of hemodilution in pregnancy on blood analyte concentrations was considered by applying a factor based on pAlb as proposed by Kaur et al.^(^ ^27^ ^)^ The mean ratio of pAlb at NPNL to that at P36 was 1.42 (SD 0.16), with no significant difference between WWH and REF (mean %Diff [95% CI]: −0.03 [−0.09, +0.03], *p =* 0.42). The effect of adjusting for hemodilution diminished the size of any increases greater than 35% (100 × log_e_[1.42–1.00]), neutralized or reversed smaller increases, and accentuated all decreases. However, such adjustments did not affect the observed group differences at P36 or changes between P36 and later timepoints and are not discussed further.

At the time of the study, Kampala, which lies close to the equator, had two wet seasons (March–May, September–December) and two dry seasons (January–February, June–August) each year, which could influence 25OHD status. Although there was evidence within individuals of higher 25OHD concentrations in wet than dry seasons (mean [95% CI] = +6.1 [+2.0, +10.1]%, *p =* 0.004, four‐timepoint model), adjustments for season had no material effect on differences between WWH and REF and are also not discussed further.

## Results

The flow of participants through the biochemical component of the Gumba Study is shown in Supplement 2 in Data S1. The biochemical data from 164 women who provided samples on at least two occasions (83 WWH, 81 REF) are presented in this paper; data from 25 participants who provided samples only at P36 (10 WWH, 15 REF) were excluded from statistical analysis. The numbers of data points per analyte in the final dataset are given in Supplement 3 in Data S1. The sensitivity analysis was conducted on the 84 participants (54 WWH, 30 REF) who provided samples at all four timepoints.

**Fig. 1:**
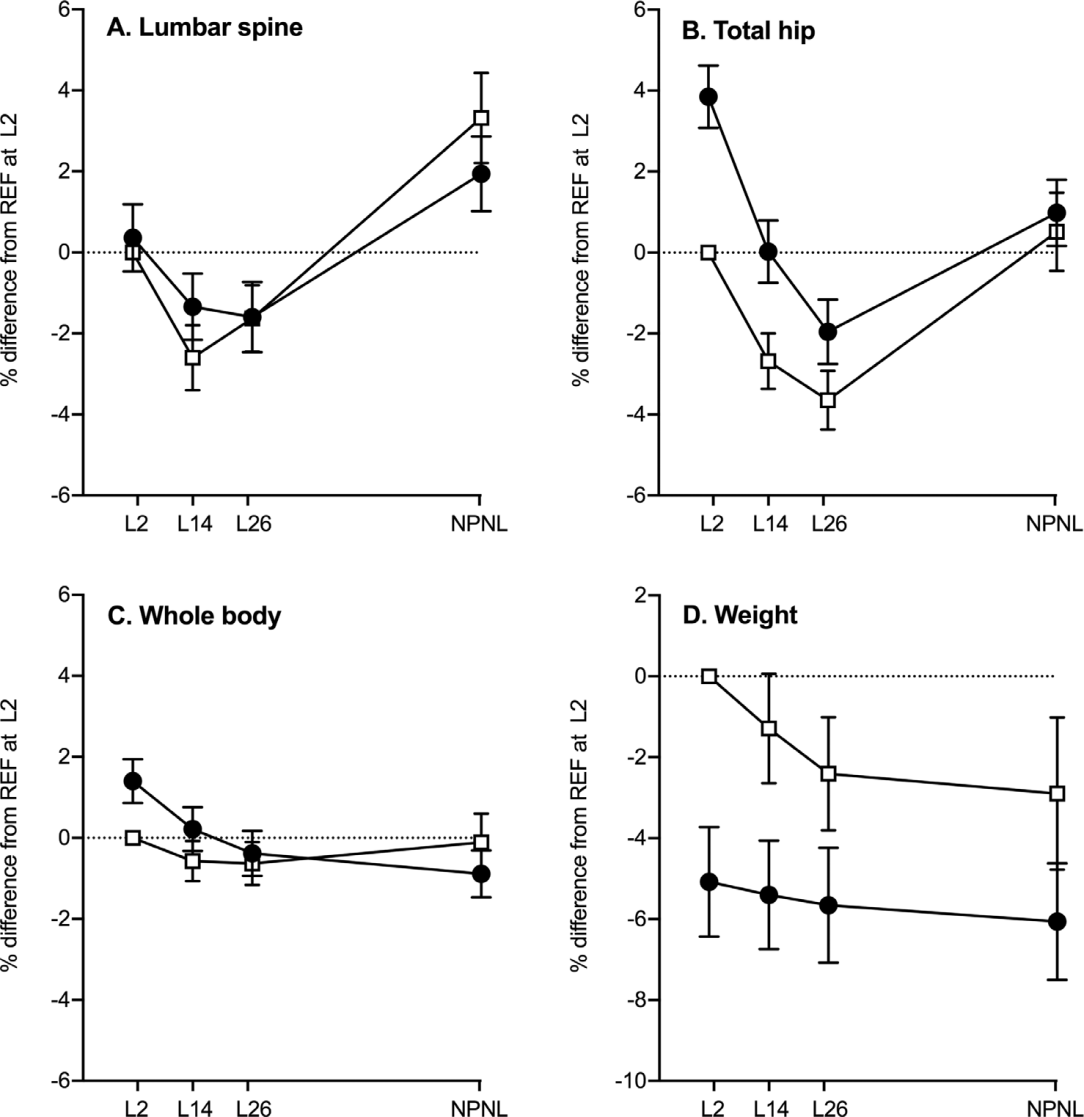
Differences in patterns of change in aBMD from 2 weeks postpartum to postlactation in two groups of Ugandan women. Data are mean percentage difference (95% CI) from REF at L2 and represent within‐group changes and between‐group differences in aBMD at the lumbar spine (*A*), total hip (*B*), and whole body (*C*), adjusted for bone area and body weight. The pattern of changes in weight (*D*) are also illustrated. Results were obtained from Scheffé post hoc tests in four‐time‐point hierarchical repeated measures ANOVA that included individual identifier (nested by group), group, timepoint, and group × timepoint interaction term with all dependent variables transformed into natural logarithms (log_e_). Black circles: WWH, women with HIV initiated on lifelong triple ART earlier in pregnancy (TDF‐3TC‐EFV, previously ART‐naïve); open squares: REF, women without HIV (reference group, ART‐naïve); L2, L14, and L26, 2, 14, and 26 weeks of lactation, respectively; NPNL, after lactation when neither pregnant nor lactating. Significance of group × timepoint interaction: lumbar spine *p* = 0.002; total hip and whole body *p* ≤ 0.0001, weight *p* = 0.24. The figure is modified from Nabwire et al.^(^ ^11^ ^)^

Table 1 presents the characteristics at each timepoint of the participants who provided blood and urine samples, and Fig. 1 illustrates the patterns of change during and after lactation in aBMD at the lumbar spine, total hip, and whole body. In summary, maternal age was comparable between groups, but fewer WWH were nulliparous at recruitment. Postpartum, more WWH exclusively breastfed for the first 6 months, but their overall duration of breastfeeding was shorter. WWH tended to have lower body weight throughout, and a greater proportion of WWH reported use of DMPA before and after the index pregnancy. As previously reported,^(^ ^11^ ^)^ both groups experienced lactational bone mineral mobilization, but WWH had greater decreases in total hip aBMD during lactation and less restitution to or above postpartum values at all three sites. Table 2 presents the biochemical data by group at each timepoint. The mean 25OHD was >50 nmol/L in both groups at all timepoints. The prevalence of 25OHD values <50 nmol/L was less than 25% in both groups, and there were only a few records of 25OHD concentration < 25 nmol/L, occurring in six WWH on one or two occasions.

**Table 1:** Characteristics and Medical History of Participants by Group and Timepoint

	P36		L14		L26		NPNL	
	WWH (*n* = 83)	REF (*n* = 81)	WWH *(n* = 83)	REF *(n* = 81)	WWH *(n* = 69)	REF *(n* = 72)	WWH *(n* = 66)	REF *(n =* 32)
Age (years)	23.6 (21.4, 27.2)	23.1 (20.8, 27.0)	24.0 (21.7, 27.6)	23.6 (21.1, 27.3)	24.1 (22.1, 27.8)	23.7 (21.4, 27.4)	24.9 (23.0, 28.8)	24.5 (22.7, 29.0)
Height (cm)	157.1 (4.6)	158.5 (5.8)	157.0 (4.4)	158.5 (5.8)	157.1 (4.4)	158.7 (5.6)	157.2 (4.6)	157.8 (4.6)
Weight (kg)	65.0 (59.2, 70.2)	66.8 (61.0, 73.0)	57.0 (53.2, 64.5)	59.9 (54.1, 67.8)	56.9 (53.0, 63.2)	59.3 (53.0, 68.7)	57.3 (51.5, 64.1)	58.7 (54.2, 67.1)
Weeks postpartum	–	–	14.3 (0.5)	14.2 (0.8)	26.4 (0.7)	26.6 (0.7)	66.8 (65.0, 71.3)***	84.5 (72.1, 93.2)
BF duration (weeks)	–	–	14.3 (0.5)	14.2 (0.8)	26.4 (0.7)	26.6 (0.7)	52.3 (47.1, 53.4)***	71.4 (55.6, 78.5)
CD_4_ count (cells/mm^3^)	396 (285, 519)	–	409 (303, 530)	–	488 (343, 674)	–	470 (338, 685)	–
Weeks on ART	10.8 (5.0)	–	29.5 (5.2)	–	42.0 (5.7)	–	80.2 (15.5)	–
Pills taken %^a^	99.1 (1.8)	–	99.7 (1.6)	–	99.4 (3.3)	–	99.2 (3.9)	–
Parity	1 (0, 2)	0 (0, 1)	2 (1, 3)	1 (1, 2)	2 (1, 3)	1 (1, 2)	2 (1, 3)	1 (1,2)
Nulli/primiparous %	36.1*	55.6	36.1*	55.6	37.7*	54.2	33.3*	53.1
EBF %	–	–	85.5***	63.0	87.0***	45.8	–	–
Resumed menses %	–	–	38.6	34.6	56.5	51.4	–	–
Current DMPA %	–	–	30.1**	13.6	36.2	20.8	43.9	31.3
Prior DMPA %	37.4	18.5	36.1**	18.5	34.8	20.8	43.9**	15.6

*Note*: Values are mean (SD) for normal distributions, median (25th, 75th percentiles) for skewed distributions, and percentage (%) for proportions of participants who reported “yes.” *, **, *** Values of *p* for difference between groups using 2‐tailed t‐test or chi‐squared test.

Abbreviations: ART = antiretroviral therapy; CD_4_ cell count = cells/mm^3^; DMPA = depot medroxyprogesterone acetate; EBF = exclusive breastfeeding; L14, L26 = 14 and 26 weeks lactation, respectively; NPNL = at least 3 months after lactation when neither pregnant nor lactating; P36 = 36 weeks of pregnancy; REF = women without HIV who provided blood samples at two or more timepoints; WWH = women with HIV initiated on tenofovir‐based ART during pregnancy (previously ART‐naïve) who provided blood samples at two or more timepoints.

^a^ Mean % adherence to ART based on pill count method used in routine clinical care = 100 × [number of pills taken/number of pills dispensed for duration].

* *p* ≤ 0.05;

** *p* ≤ 0.01;

*** *p* ≤ 0.001.

**Table 2:** Biochemical Markers of Bone Turnover and Mineral Metabolism by Group at Each Timepoint^a^

	P36		L14		L26		NPNL	
	WWH (*n* = 83)	REF (*n* = 81)	WWH *(n* = 83)	REF *(n* = 81)	WWH *(n* = 69)	REF *(n* = 72)	WWH *(n* = 66)	REF *(n =* 32)
Bone turnover markers								
CTX ng/L	505 (362, 724)	529 (429, 659)	1063 (898, 1477)^b^	915 (713, 1246)	982 (717, 1488)^b^	841 (673, 1227)	411 (292, 617)	366 (216, 553)
P1NP μg/L	55.7 (38.3, 82.2)^b^ ^,^ ***	72.4 (52.5, 105)	151 (117, 197)	151 (124, 186)	137 (110, 168)	142 (117, 184)	109 (80.0, 161)^b^ ^,^ *	91.6 (53.0, 146)
BALP μg/L	20.4 (16.0, 25.2)	19.3 (14.8, 24.8)	40.3 (31.3, 53.4)^b^ ^,^ ***	34.2 (26.9, 44.3)	48.8 (39.0, 62.1)^b^ ^,^ ***	40.6 (32.2, 53.2)	34.8 (27.7, 43.4)^b^ ^,^ ***	25.9 (16.9, 42.7)
TALP U/L	148 (117, 185)	152 (117, 196)	141 (112, 171)^b^ ^,^ ***	117 (98, 138)	138 (115, 164)^b^ ^,^ **	122 (100, 154)	120 (106, 142)^b^ ^,^ ***	92.5 (67.0, 135)
P1NP/CTX 1000 × μg/ng	110 (86, 133)^b^ ^,^ **	136 (103, 172)	142 (97, 197)^b^	165 (123, 219)	140 (108, 180)^b^	169 (132, 220)	266 (191, 345)	250 (175, 355)
Hormones and vitamin D status								
PTH ng/L	32.6 (24.1, 44.9)^b^ ^,^ ***	23.2 (15.8, 35.8)	59.0 (44.7, 80.0)^b^ ^,^ ***	41.3 (29.5, 58.8)	61.0 (44.3, 80.0)^b^ ^,^ ***	45.4 (34.1, 63.2)	54.0 (41.4, 68.2)^b^	39.0 (27.8, 51.9)
FGF23 ng/L	5.07 (3.11, 6.86)	4.33 (3.02, 5.61)	10.7 (7.58, 13.6)	12.3 (8.81, 14.7)	10.5 (7.78, 12.5)^b^	12.9 (9.7, 15.4)	9.92 (7.26, 11.2)	9.22 (6.77, 11.9)
1,25(OH)_2_D pmol/L	258 (214, 304)^b^ ^,^ **	292 (259, 331)	178 (151, 209)	192 (153, 237)	177 (152, 202)	188 (166, 212)	170 (143, 211)^b^	191 (166, 226)
25OHD nmol/l	67.7 (53.4, 90.6)	65.0 (54.6, 79.3)	65.9 (54.4, 95.6)^b^ ^,^ *	59.2 (50.6, 71.1)	63.7 (51.4, 76.8)	67.9 (58.0, 83.2)	60.2 (51.5, 78.1)^b^	68.3 (57.1, 81.7)
Plasma chemistry								
pAlb g/L	26.3 (24.8, 28.3)	26.6 (25.2, 28.2)	37.7 (35.8, 39.6)^b^	38.7 (36.8, 40.8)	37.9 (36.2, 40.3)	37.4 (35.4, 40.0)	36.9 (35.7. 38.6)	37.8 (36.2, 39.5)
pCa mmol/L	2.17 (2.11, 2.24)^b^	2.20 (2.15, 2.27)	2.23 (2.16, 2.32)^b^ ^,^ ***	2.36 (2.30, 2.43)	2.20 (2.12, 2.29)	2.19 (2.12, 2.27)	2.35 (2.29, 2.42)^b^	2.40 (2.36, 2.46)
pCa_corr_ mmol/L	2.44 (2.39, 2.51)^b^	2.47 (2.42, 2.52)	2.28 (2.21, 2.36)^b^ ^,^ ***	2.38 (2.33, 2.44)	2.24 (2.18, 2.29)	2.24 (2.18, 2.30)	2.41 (2.37, 2.48)	2.44 (2.39, 2.51)
pP mmol/L	1.10 (0.99, 1.23)^b^ ^,^ ***	1.22 (1.10, 1.33)	1.27 (1.15, 1.44)	1.31 (1.21, 1.45)	1.18 (1.09, 1.30)	1.21 (1.09, 1.36)	1.13 (1.02, 1.26)	1.15 (1.04, 1.28)
pMg mmol/L	0.74 (0.70, 0.79)	0.74 (0.70, 0.77)	0.79 (0.76, 0.83)	0.80 (0.75, 0.84)	0.77 (0.74, 0.81)	0.78 (0.75, 0.82)	0.83 (0.78, 0.87)	0.83 (0.81, 0.86)
pCr μmol/L	42.5 (37.4, 48.9)	41.5 (34.8, 48.8)	58.0 (51.5, 66.7)	55.1 (49.6, 59.9)	54.0 (48.2, 60.2)^b^ ^,^ *	49.5 (45.1, 54.7)	62.7 (56.4, 71.3)	60.8 (56.6, 65.0)
Urine chemistry and renal function								
eGFR ml/min/1.73m^2^	137 (132, 144)	138 (130, 147)	119 (112, 129)^b^ ^,^ **	124 (120, 131)	124 (121, 131)^b^ ^,^ **	130 (124, 136)	111 (101, 125)	117 (112, 127)
TmCa/GFR mmol/L	2.76 (2.31, 3.29)	2.73 (2.36, 2.98)	3.30 (2.70, 4.01)	3.28 (2.67, 3.87)	3.15 (2.78, 3.72)	3.03 (2.60, 3.61)	3.41 (3.13, 3.80)	3.49 (3.08, 4.01)
TmP/GFR mmol/L	1.28 (1.10, 1.50)^b^ ^,^ ***	1.47 (1.28, 1.68)	1.50 (1.36, 1.72)^b^	1.59 (1.47, 1.77)	1.37 (1.23, 1.53)^b^ ^,^ *	1.49 (1.35, 1.68)	1.29 (1.13, 1.52)^b^	1.37 (1.18, 1.66)
2 h uCa/Cr 100 × mmol/mmol	3.01 (1.43, 6.71)	3.51 (1.78, 8.56)	1.09 (0.46, 2.34)^b^	1.61 (0.63, 4.24)	1.25 (0.55, 2.32)	1.64 (0.64, 3.05)	1.05 (0.61, 1.63)	1.14 (0.74, 1.78)
2 h uP/Cr mmol/mmol	1.27 (0.95, 1.72)	1.23 (0.96, 1.57)	1.02 (0.77, 1.57)	0.91 (0.64, 1.51)	1.09 (0.75, 1.62)^b^	0.83 (0.53, 1.47)	0.93 (0.62, 1.33)^b^	0.79 (0.46, 1.30)
2 h uMg/Cr mmol/mmol	0.18 (0.14, 0.26)	0.20 (0.14, 0.27)	0.19 (0.14, 0.28)	0.17 (0.12, 0.26)	0.21 (0.14, 0.33)^b^ ^,^ *	0.17 (0.13, 0.26)	0.21 (0.15, 0.30)	0.18 (0.13, 0.26)

*Note*: Data are geometric means (25th, 75th percentiles); the number of data points per analyte by group and timepoint are given in Supplement 3 in Data S1. *, **, *** Values of *p* for difference between the groups from Scheffé post hoc tests for group × timepoint interaction terms in four‐time‐point hierarchical repeated‐measures ANOVA models, which included participant ID (nested by group), group, timepoint, and group × timepoint interaction. Variables were transformed into natural logarithms and multiplied by 100 before data analysis.

Abbreviations: 1,25(OH)_2_D = 1,25‐dihydroxyvitamin D; 2 h uCa/Cr = 2 h fasting ratio of urinary calcium to creatinine; 2 h uMg/Cr = 2 h fasting ratio of urinary magnesium to creatinine; 2 h uP/Cr = 2 h fasting ratio of urinary phosphorus to creatinine; 25OHD = 25‐hydroxyvitamin D; ART = antiretroviral therapy; BALP = bone‐specific alkaline phosphatase; CTX = C‐terminal telopeptide; eGFR = estimated glomerular filtration rate (CKD); FGF23 = intact fibroblast growth factor‐23; L14, L26 = 14, 26 weeks lactation respectively; NPNL = at least 3 months post‐lactation when neither pregnant nor lactating; P1NP = procollagen type 1 N‐terminal propeptide; P1NP/CTX = ratio of P1NP to CTX; P36 = 36 weeks of pregnancy; pAlb = plasma albumin; pCa = plasma calcium; pCa_corr_ = albumin corrected plasma calcium (Payne); pCr = plasma creatinine; pMg = plasma magnesium; pP = plasma phosphate; PTH = parathyroid hormone; REF = women without HIV who provided blood samples at 2 or more timepoints; TALP = total alkaline phosphatase; TmCa/GFR = renal tubular maximum reabsorption of calcium per unit volume of glomerular filtrate; TmP/GFR = renal tubular maximum reabsorption of phosphate per unit volume of glomerular filtrate; WWH = women with HIV initiated on tenofovir‐based ART during pregnancy (previously ART‐naïve) who provided blood samples at 2 or more timepoints.

^a^ The number of data points per analyte by group and timepoint are given in Supplement 3 in Data S1.

^b^ Significant group difference in cross‐sectional models with adjustment for predictors (mean percentage difference (95% CI) and *p* values from these models are in Supplement 4 in Data S1).

* *p* ≤ 0.05;

** *p* ≤ 0.01;

*** *p* ≤ 0.001.

**Fig. 2:**
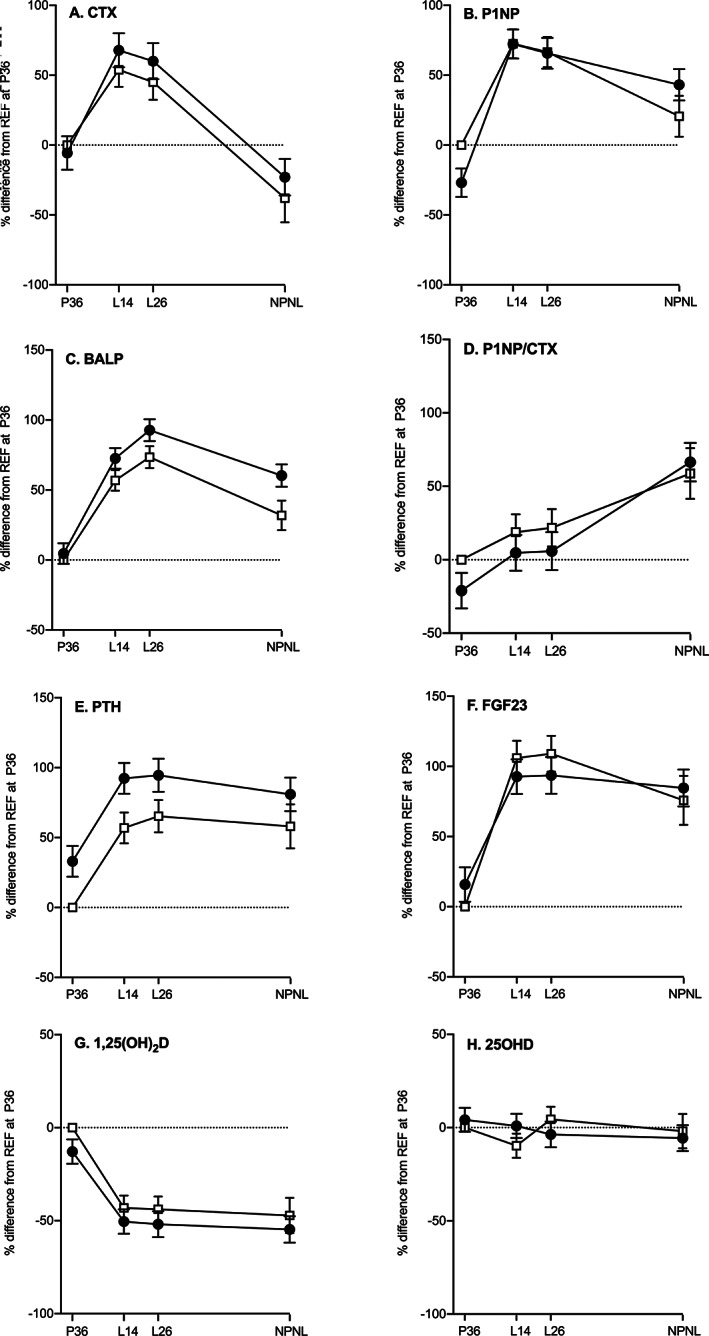
Differences in patterns of change in bone turnover markers and calciotropic hormones from late pregnancy to postlactation in two groups of Ugandan women. Data are mean percentage difference (95% CI) from REF at P36 and represent within‐group changes and between‐group differences for these blood‐borne analytes. Results were obtained from Scheffé post hoc tests in four‐time‐point hierarchical repeated measures ANOVA that included individual identifier (nested by group), group, timepoint, and group × timepoint interaction term with all dependent variables transformed into natural logarithms (log_e_). Black circles: WWH, women with HIV initiated on lifelong triple ART earlier in pregnancy (TDF‐3TC‐EFV, previously ART‐naïve); open squares: REF, women without HIV (reference group, ART‐naïve); P36, 36 weeks of gestation; L14 and L26, 14 and 26 weeks of lactation, respectively; NPNL, after lactation when neither pregnant nor lactating. (*A*) CTX, C‐terminal telopeptide (bone resorption marker); (*B*) P1NP, procollagen type 1 N‐terminal propeptide (bone formation marker); (*C*) BALP, bone‐specific alkaline phosphatase (bone formation marker); (*D*) P1NP/CTX, ratio of P1NP to CTX (index of balance between bone formation and resorption); (*E*) PTH, parathyroid hormone; (*F*) FGF23, intact fibroblast growth factor‐23; (*G*) 1,25(OH)_2_D, 1,25‐dihydroxyvitamin D; (*H*) 25OHD, 25‐hydroxyvitamin D. Data for differences between WWH and REF can be found in Table 5 and the significance of differences in the patterns of change over time in Supplement 5 in Data S1.

**Fig. 3:**
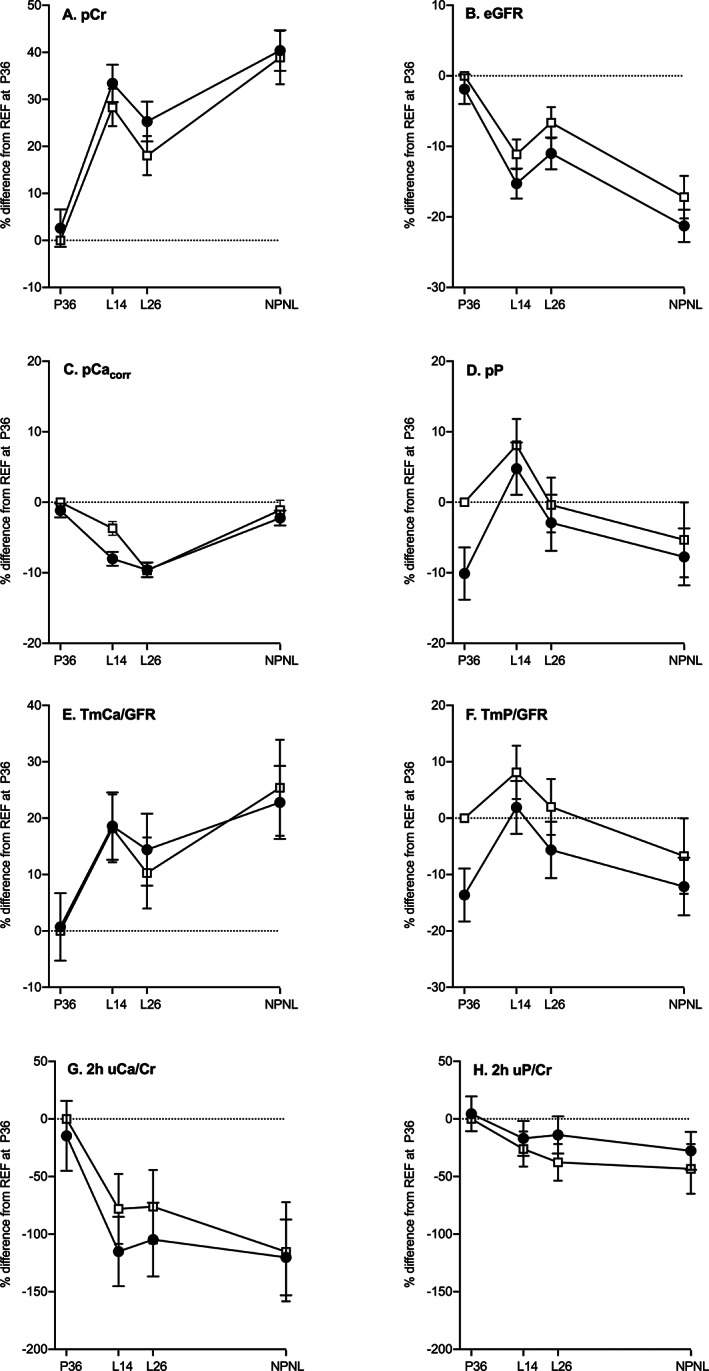
Differences in patterns of change in markers of calcium and phosphate metabolism and renal function from late pregnancy to after lactation in two groups of Ugandan women. Data are mean percentage difference (95% CI) from REF at P36 and represent within‐group changes and between‐group differences for these blood‐borne and urinary analytes. Results were obtained from Scheffé post hoc tests in four‐time‐point hierarchical repeated measures ANOVA that included individual identifier (nested by group), group, timepoint, and group × timepoint interaction term with all dependent variables transformed into natural logarithms (log_e_). Black circles: WWH, women with HIV initiated on lifelong triple ART earlier in pregnancy (TDF‐3TC‐EFV, previously ART‐naïve); open squares: REF, women without HIV (reference group, ART‐naïve); P36, 36 weeks of gestation; L14 and L26, 14 and 26 weeks of lactation, respectively; NPNL, after lactation when neither pregnant nor lactating. (*A*) pCr, plasma creatinine; (*B*) eGFR, estimated glomerular filtration rate (CKD); (*C*) pCa_corr_, albumin‐corrected plasma calcium (Payne); (*D*) pP, plasma phosphate; (*E*) TmCa/GFR, renal tubular maximum reabsorption of calcium per unit volume of glomerular filtrate; (*F*) TmP/GFR renal tubular maximum reabsorption of phosphate per unit volume of glomerular filtrate; (*G*) 2 h uCa/Cr, 2 h fasting ratio of urinary calcium to creatinine; (*H*) 2 h uP/Cr, 2 h fasting ratio of urinary phosphorus to creatinine. Data for differences between WWH and REF can be found in Table 5 and the significance of differences in the patterns of change over time in Supplement 5 in Data S1.

**Fig. 4:**
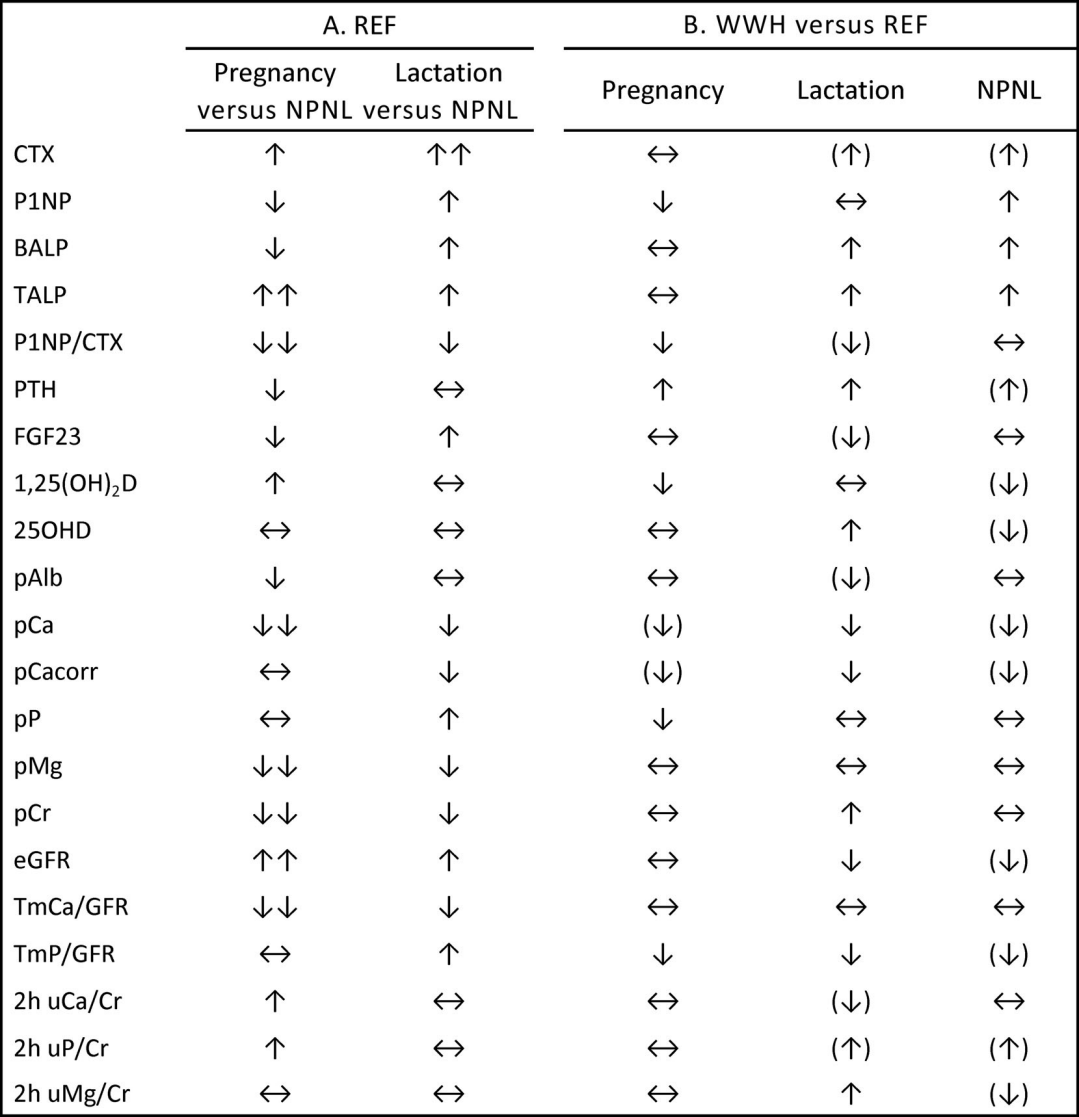
Schematic diagram depicting (*A*) the within‐individual biochemical profile of reference women (REF) in late pregnancy (P36) and 14–26 weeks of lactation (L14, L26) compared to after lactation when neither pregnant nor lactating (NPNL) and (*B*) the pattern of biochemical differences between women with HIV (WWH) and REF at each of these three life stages. Solid arrow: significant difference (higher or lower) in hierarchical and regression models; in lactation, this represents a difference that was significant either at L14 or L26, or both. Solid horizontal arrows: small difference that was not significant from zero in any model; in lactation, this represents a difference that was not significant at both L14 and L26. Double solid arrows in same direction (section A only): significant difference (higher or lower) in any model in same direction but of significantly greater magnitude than in the preceding or subsequent timepoint; solid arrow in parentheses (section B only): group difference that was not significant in hierarchical models but was either significant in adjusted cross‐sectional models or of a similar magnitude to timepoints where the difference was significant. CTX, C‐terminal telopeptide (bone resorption marker); P1NP, procollagen type 1 N‐terminal propeptide (bone formation marker); BALP and TALP, bone‐specific and total alkaline phosphatase (bone formation markers); P1NP/CTX, ratio of P1NP to CTX (index of balance between bone formation and resorption); PTH, parathyroid hormone; FGF23, intact fibroblast growth factor‐23; 1,25(OH)_2_D, 1,25‐dihydroxyvitamin D; 25OHD, 25‐hydroxyvitamin D; pAlb = plasma albumin; pCa = plasma calcium; pCa_corr_ = albumin‐corrected plasma calcium (Payne); pP = plasma phosphate; pMg = plasma magnesium; pCr = plasma creatinine; eGFR = estimated glomerular filtration rate (CKD); TmCa/GFR = renal tubular maximum reabsorption of calcium per unit volume of glomerular filtrate; TmP/GFR = renal tubular maximum reabsorption of phosphate per unit volume of glomerular filtrate; 2 h uCa/Cr = 2 h fasting ratio of urinary calcium to creatinine; 2 h uP/Cr = 2 h fasting ratio of urinary phosphorus to creatinine; 2 h uMg/Cr = 2 h fasting ratio of urinary magnesium to creatinine.

The patterns of change in the biochemical markers relative to REF at P36 are illustrated by group for key analytes in Figs. 2 and 3. The direction of biochemical changes in REF during pregnancy and lactation relative to NPNL are summarized in the schematic diagram (Fig. 4*A*) and the direction of differences between WWH and REF at each timepoint is summarized in Fig. 4*B*. Table 3 presents the data by group on within‐individual changes in the biochemical markers between timepoints for those relating to changes from pregnancy into and across lactation, and Table 4 presents those for changes from pregnancy and lactation to NPNL. The differences between WWH and REF at each timepoint from the four‐timepoint models are presented in Table 5, and those from cross‐sectional regression models with adjustment for significant predictor variables are provided in Supplement 4 in Data S1. In Tables 2, 3, 4, 5 the significance of changes between timepoints and differences between groups are indicated as *p* values from the Scheffé tests in the four‐timepoint models. The *p* values of group × timepoint interaction terms in the four‐, three‐, and two‐timepoint models are given in Supplement 5 in Data S1. Restricting statistical models to those participants with data at all timepoints provided estimates that were comparable to results obtained with the full dataset (Supplements 6–11 in Data S1).

**Table 3:** Within‐Individual Changes in Biochemical Markers by Group from Pregnancy to Lactation and During Lactation^a^

	P36 to L14				L14 to L26			
	WWH %∆ (95% CI)	*p*	REF %∆ (95% CI)	*p*	WWH %∆ (95% CI)	*p*	REF %∆ (95% CI)	*p*
Bone turnover markers								
CTX	+73.4 (+61.4, +85.4)	<0.0001	+53.9 (+41.7, +66.1)	<0.0001	−7.8 (−20.5, + 4.9)	0.70	−8.8 (−21.3, +3.7)	0.59
P1NP	+99.2 (+89.0, +109.4)	<0.0001	+72.5 (+62.1, +82.9)	<0.0001	−6.6 (−17.6, +4.4)	0.71	−5.9 (−16.7, +4.9)	0.76
BALP	+67.8 (+60.4, +75.2)	<0.0001	+56.8 (+49.4, +64.2)	<0.0001	+20.2 (+12.4, +28.0)	<0.0001	+16.6 (+8.8, +24.4)	<0.0001
TALP	−4.2 (−11.3, +2.9)	0.72	−25.8 (−33.1, −18.5)	<0.0001	−1.3 (−8.9, +6.3)	0.99	+3.3 (−4.3, +10.9)	0.87
P1NP/CTX	+25.8 (+13.6, +38.0)	0.0007	+18.8 (+6.6, +31.0)	0.03	+1.2 (−11.7, +14.1)	0.99	+3.0 (−9.7, +15.7)	0.98
Hormones and vitamin D status								
PTH	+59.3 (+48.3, +70.3)	<0.0001	+56.9 (+45.9, +67.9)	<0.0001	+2.2 (−9.6, +14.0)	0.99	+8.5 (−3.1, +20.1)	0.56
FGF23	+76.9 (+64.6, +89.2)	<0.0001	+106.0 (+93.7. +118.3)	<0.0001	+1.0 (−12.1, +14.1)	0.99	+3.1 (−7.6, +17.8)	0.97
1,25(OH)_2_D	−37.6 (−44.1, −31.1)	<0.0001	−43.1 (−49.8, −36.4)	<0.0001	−1.4 (−8.3, +5.5)	0.98	−0.8 (−7.7, +6.1)	0.99
25OHD	−3.3 (−9.8, +3.2)	0.80	−9.7 (−16.2, −3.2)	0.04	−4.6 (−11.5, +2.3)	0.64	+14.2 (7.3, +21.1)	<0.0001
Plasma chemistry								
pAlb	+35.8 (+33.8, +37.8)	<0.0001	+37.6 (+35.6, +39.6)	<0.0001	+0.6 (−1.6, +2.8)	0.96	−3.2 (−5.4, −1.0)	0.04
pCa	+2.9 (+1.7, +4.1)	<0.0001	+6.7 (+5.5, +7.9)	<0.0001	−1.7 (−2.9, −0.5)	0.09	−7.1 (−8.3, −5.9)	<0.0001
pCa_corr_	−6.8 (−7.8, −5.8)	<0.0001	−3.7 (−4.7, −2.7)	<0.0001	−1.6 (−2.6, −0.6)	0.03	−6.0 (−7.0, −5.0)	<0.0001
pP	+14.9 (+11.2, +18.6)	<0.0001	+8.1 (+4.4, +11.8)	0.0005	−7.6 (−11.5, −3.7)	0.003	−8.5 (−12.4, −4.6)	0.0005
pMg	+6.9 (+4.9, +8.9)	<0.0001	+8.1 (+6.1, +10.1)	<0.0001	−2.9 (−4.9, −0.9)	0.05	−1.5 (−3.5, + 0.5)	0.53
pCr	+30.7 (+26.8, +34.6)	<0.0001	+28.3 (+24.4, +32.2)	<0.0001	−8.1 (−12.4, −3.8)	0.003	−10.3 (−14.4, −6.2)	<0.0001
Urine chemistry and renal function								
eGFR	−13.4 (−15.6, −11.2)	<0.0001	−11.1 (−13.3, −8.9)	<0.0001	+4.3 (+1.9, +6.7)	0.003	+4.5 (+2.3, +6.7)	0.001
TmCa/GFR	+17.9 (+12.0, +23.8)	<0.0001	+18.2 (+12.1, +24.3)	<0.0001	−4.1 (−10.6, +2.4)	0.65	−7.9 (−14.2, −1.6)	0.11
TmP/GFR	+15.5 (+10.8, +20.2)	<0.0001	+8.1 (+3.4, +12.8)	0.01	−7.5 (−12.4, −2.6)	0.03	−6.1 (−11.0, −1.2)	0.12
2 h uCa/Cr	−100.4 (−130.6, −70.2)	<0.0001	−78.1 (−108.5, −47.7)	<0.0001	+10.5 (−21.6, +42.6)	0.94	+1.9 (−30.0, +33.8)	0.99
2 h uP/Cr	−21.3 (−36.4, −6.2)	0.05	−26.2 (−41.5, −10.9)	0.01	+3.0 (−13.1, +19.1)	0.99	−11.5 (−27.6, +4.6)	0.57
2 h uMg/Cr	+4.5 (−9.2, +18.2)	0.94	−12.3 (−26.2, +1.6)	0.39	+12.5 (−2.2, +27.2)	0.42	+0.6 (−14.1, +15.3)	0.99

*Note*: Values are within‐individual mean percentage changes (%∆) and 95% confidence intervals (95% CIs) obtained from Scheffé post hoc tests for group × timepoint interaction terms in four‐time‐point hierarchical repeated‐measures ANOVA models, which included participant ID (nested by group), group, timepoint, and group × timepoint interaction. Variables were transformed into natural logarithms and multiplied by 100 before data analysis. The plus or minus signs show the direction of within‐group changes (increase or decrease, respectively).

Abbreviations: 1,25(OH)_2_D = 1,25‐dihydroxyvitamin D (pmol/L); 2 h uCa/Cr = 2 h fasting ratio of urinary calcium to creatinine 100×mmol/mmol; 2 h uMg/Cr = 2 h fasting ratio of urinary magnesium to creatinine (mmol/mmol); 2 h uP/Cr = 2 h fasting ratio of urinary phosphorus to creatinine (mmol/mmol); 25OHD = 25‐hydroxyvitamin D (nmol/L); ART = antiretroviral therapy; BALP = bone‐specific alkaline phosphatase (μg/L); CTX = C‐terminal telopeptide (ng/L); eGFR = estimated glomerular filtration rate (CKD, ml/min/1.73 m^2^); FGF23 = intact fibroblast growth factor‐23 (ng/L); L14, L26 = 14, 26 weeks lactation, respectively; P1NP = procollagen type 1 N‐terminal propeptide (μg/L); P1NP/CTX = ratio of P1NP to CTX (1000×μg/ng); P36 = 36 weeks of pregnancy; pAlb = plasma albumin (g/L); pCa = plasma calcium (mmol/L); pCacorr = albumin‐corrected plasma calcium (Payne, mmol/L); pCr = plasma creatinine (μmol/L); pMg = plasma magnesium (mmol/L); pP = plasma phosphate (mmol/L); PTH = parathyroid hormone (ng/L); REF = women without HIV who provided blood samples at two or more timepoints; TALP = total alkaline phosphatase U/L; TmCa/GFR = renal tubular maximum reabsorption of calcium per unit volume of glomerular filtrate (mmol/L); TmP/GFR = renal tubular maximum reabsorption of phosphate per unit volume of glomerular filtrate (mmol/L); WWH = women with HIV initiated on tenofovir‐based ART during pregnancy (previously ART‐naïve) who provided blood samples at two or more timepoints.

^a^ The number of data points per analyte by group and timepoint are given in Supplement 3 in Data S1.

**Table 4:** Within‐Individual Changes in Biochemical Markers to 3 Months After Lactation by Group^a^

	L26 to NPNL				P36 to NPNL			
	WWH %∆ (95% CI)	*p*	REF %∆ (95% CI)	*p*	WWH %∆ (95% CI)	*p*	REF %∆ (95% CI)	*p*
Bone turnover markers								
CTX	−83.2 (−96.7, −69.7)	<0.0001	−83.1 (−100.5, −65.7)	<0.0001	−17.5 (−30.4, −4.6)	0.07	−38.0 (−55.4, −20.6)	0.0004
P1NP	−22.5 (−34.1, −10.9)	0.002	−46.0 (−60.9, −31.1)	<0.0001	+70.1 (+59.1, +81.1)	<0.0001	+20.6 (+5.9, +35.3)	0.06
BALP	−32.4 (−40.8, −24.0)	<0.0001	−41.6 (−52.4, −30.8)	<0.0001	+55.7 (+47.7, +63.7)	<0.0001	+31.8 (+21.2, +42.4)	<0.0001
TALP	−12.9 (−21.1, −4.7)	0.02	−26.0 (−36.4, −15.6)	<0.0001	−18.4 (−26.2, −10.6)	0.0001	−48.5 (−58.7, −38.3)	<0.0001
P1NP/CTX	+60.6 (+47.1, +74.1)	<0.0001	+37.0 (+19.6, +54.4)	0.0008	+87.6 (+74.7, +100.5)	<0.0001	+58.8 (+41.6, +76.0)	<0.0001
Hormones and vitamin D status								
PTH	−13.6 (−25.9, −1.3)	0.20	−7.4 (−23.3, +8.5)	0.84	+47.9 (+36.1, +59.7)	<0.0001	+58.0 (+42.3, +73.7)	<0.0001
FGF23	−9.0 (−22.7, +4.7)	0.65	−33.3 (−51.1, −15.5)	0.004	+68.9 (+55.8, +82.0)	<0.0001	+75.8 (+58.2, +93.4)	<0.0001
1,25(OH)_2_D	−2.7 (−10.1, +4.7)	0.91	−3.3 (−12.9, +6.3)	0.93	−41.8 (−48.9, −34.7)	<0.0001	−47.2 (−56.8, −37.6)	<0.0001
25OHD	−1.9 (−9.2, +5.4)	0.97	−6.3 (−15.7, +3.1)	0.62	−9.8 (−16.7, −2.9)	0.06	−1.9 (−11.1, +7.3)	0.98
Plasma chemistry								
pAlb	−2.3 (−4.7, +0.1)	0.30	+1.7 (−1.2, +4.6)	0.74	+34.1 (+31.9, +36.3)	<0.0001	+36.1 (+33.2, +39.0)	<0.0001
pCa	+7.0 (+5.6, +8.4)	<0.0001	+9.2 (+7.4, +11.0)	<0.0001	+8.2 (+7.0, +9.4)	<0.0001	+8.8 (+7.0, +10.6)	<0.0001
pCa_corr_	+7.4 (+6.2, +8.6)	<0.0001	+8.6 (+7.2, +10.0)	<0.0001	−1.1 (−2.1, −0.1)	0.27	−1.1 (−2.5, +0.3)	0.48
pP	−4.9 (−9.0, −0.8)	0.16	−4.9 (−10.4, +0.6)	0.36	+2.4 (−1.5, +6.3)	0.72	−5.3 (−10.6, 0.0)	0.28
pMg	+6.3 (+4.1, +8.5)	<0.0001	+6.0 (+3.3, +8.7)	0.0004	+10.3 (+8.3, +12.3)	<0.0001	+12.6 (+9.9, +15.3)	<0.0001
pCr	+15.2 (+10.7, +19.7)	<0.0001	+20.9 (+15.0, +26.8)	<0.0001	+37.8 (+33.5, +42.1)	<0.0001	+38.9 (+33.2, +44.6)	<0.0001
Urine chemistry and renal function								
eGFR	−10.3 (−12.7, −7.9)	<0.0001	−10.6 (−13.7, −7.5)	<0.0001	−19.4 (−21.8, −17.0)	<0.0001	−17.2 (−20.1, −14.3)	<0.0001
TmCa/GFR	+8.4 (+1.5, +15.3)	0.12	+15.1 (+6.5, +23.7)	0.009	+22.1 (+15.6, +28.6)	<0.0001	+25.4 (+16.8, +34.0)	<0.0001
TmP/GFR	−6.5 (−11.8, −1.2)	0.13	−8.7 (−15.6, −1.8)	0.10	+1.5 (−3.6, +6.6)	0.95	−6.7 (−13.4, 0.0)	0.28
2 h uCa/Cr	−15.5 (−49.8, +18.8)	0.85	−39.1 (−83.0, +4.8)	0.39	−105.5 (−138.4, −72.6)	<0.0001	−115.3 (−158.4, −72.2)	<0.0001
2 h uP/Cr	−13.8 (−31.0, +3.4)	0.49	−5.6 (−27.6, +16.4)	0.97	−32.1 (−48.6, −15.6)	0.003	−43.4 (−65.0, −21.8)	0.002
2 h uMg/Cr	−1.2 (−16.9, +14.5)	0.99	−0.1 (−20.1, +19.9)	0.99	+15.8 (+0.9, +30.7)	0.24	−11.8 (−31.4, +7.8)	0.71

*Note*: Values are within‐individual mean percentage changes (%∆) and 95% confidence intervals (95% CIs) obtained from Scheffé post hoc tests for group×timepoint interaction terms in four‐time‐point hierarchical repeated‐measures ANOVA models, which included participant ID (nested by group), group, timepoint, and group×timepoint interaction. Variables were transformed into natural logarithms and multiplied by 100 before data analysis. The plus and minus signs show the direction of within‐group changes (increase or decrease, respectively).

Abbreviations: 1,25(OH)_2_D = 1,25‐dihydroxyvitamin D (pmol/L); 2 h uCa/Cr = 2 h fasting ratio of urinary calcium to creatinine 100×mmol/mmol; 2 h uMg/Cr = 2 h fasting ratio of urinary magnesium to creatinine (mmol/mmol); 2 h uP/Cr = 2 h fasting ratio of urinary phosphorus to creatinine (mmol/mmol); 25OHD = 25‐hydroxyvitamin D (nmol/L); ART = antiretroviral therapy; BALP = bone‐specific alkaline phosphatase (μg/L); CTX = C‐terminal telopeptide (ng/L); eGFR = estimated glomerular filtration rate (CKD, ml/min/1.73 m^2^); FGF23 = intact fibroblast growth factor‐23 (ng/L); L26 = 26 weeks lactation; NPNL = at least 3 months after lactation when neither pregnant nor lactating; P1NP = procollagen type 1 N‐terminal propeptide (μg/L); P1NP/CTX = ratio of P1NP to CTX (1000 × μg/ng); P36 = 36 weeks of pregnancy; pAlb = plasma albumin (g/L); pCa = plasma calcium (mmol/L); pCacorr = albumin‐corrected plasma calcium (Payne, mmol/L); pCr = plasma creatinine (μmol/L); pMg = plasma magnesium (mmol/L); pP = plasma phosphate (mmol/L); PTH = parathyroid hormone (ng/L); REF = women without HIV who provided blood samples at two or more timepoints; TALP = total alkaline phosphatase U/L; TmCa/GFR = renal tubular maximum reabsorption of calcium per unit volume of glomerular filtrate (mmol/L); TmP/GFR = renal tubular maximum reabsorption of phosphate per unit volume of glomerular filtrate (mmol/L); WWH = women with HIV initiated on tenofovir‐based ART during pregnancy (previously ART‐naïve) who provided blood samples at two or more timepoints.

^a^ The number of data points per analyte by group and timepoint are given in Supplement 3 in Data S1.

**Table 5:** Summary of Percentage Differences Between WWH and REF at Each Timepoint^a^

	P36		L14		L26		NPNL	
	%Diff (95% CI)	*p*	%Diff (95% CI)	*p*	%Diff (95% CI)	*p*	%Diff (95% CI)	*p*
Bone turnover markers								
CTX	−5.6 (−17.6, +6,4)	0.84	+13.9 (+1.7, +26.1)	0.16^b^	+15.0 (+1.7, +28.3)	0.18^b^	+15.0 (−2.8, +32.8)	0.44
P1NP	−26.9 (−37.1, −16.7)	<0.0001^b^	−0.2 (−10.6, +10.2)	0.99	−0.8 (−12.2, +10.6)	0.99	+22.6 (+7.3, +37.9)	0.04^b^
BALP	+4.6 (−2.8, +12.0)	0.68	+15.7 (+8.3, +23.1)	0.0008^b^	+19.3 (+11.1, +27.5)	0.0001^b^	+28.5 (+17.3, +39.7)	<0.0001^b^
TALP	−3.1 (−10.4, +4.2)	0.87	+18.5 (+11.2, +25.8)	<0.0001^b^	+13.9 (+5.9, +21.9)	0.009^b^	+27.1 (+16.3, +37.9)	<0.0001^b^
P1NP/CTX	−21.1 (−33.3, −8.9)	0.009^b^	−14.1 (−26.3, −1.9)	0.16^b^	−15.9 (−29.2, −2.6)	0.15^b^	+7.7 (−10.3, +25.7)	0.87
Hormones and vitamin D status								
PTH	+33.0 (+22.0, +44.0)	<0.0001^b^	+35.4 (+24.4, +46.4)	<0.0001^b^	+29.2 (+17.0, +41.4)	<0.0001^b^	+22.9 (+6.6, +39.2)	0.06^b^
FGF23	+15.8 (+3.5, +28.1)	0.10	−13.3 (−25.6, −1.0)	0.22	−15.5 (−29.0, −2.0)	0.18^b^	+8.8 (−9.4, +27.0)	0.82
1,25(OH)_2_D	−12.9 (−19.6, −6.2)	0.002^b^	−7.5 (−14.0, −1.0)	0.17	−8.1 (−15.4, −0.8)	0.19	−7.5 (−17.3, +2.3)	0.53^b^
25OHD	+4.2 (−2.3, +10.7)	0.65	+10.6 (+4.1, +17.1)	0.02^b^	−8.1 (−15.2, −1.0)	0.17	−3.7 (−13.3, +5.9)	0.90^b^
Plasma chemistry								
pAlb	−1.0 (−3.0, +1.0)	0.83	−2.8 (−4.8, −0.8)	0.06^b^	+1.0 (−1.4, +3.4)	0.87	−3.0 (−5.9, −0.1)	0.30
pCa	−1.5 (−2.7, −0.3)	0.12^b^	−5.3 (−6.5, −4.1)	<0.0001^b^	+0.2 (−1.2, +1.6)	0.99	−2.1 (−3.9, −0.3)	0.15^b^
pCa_corr_	−1.2 (−2.2, −0.2)	0.14^b^	−4.3 (−5.3, −3.3)	<0.0001^b^	+0.1 (−1.1, +1.3)	0.99	−1.1 (−2.5, +0.3)	0.51
pP	−10.1 (−13.8, −6.4)	<0.0001^b^	−3.3 (−7.0, +0.4)	0.38	−2.5 (−6.6, +1.6)	0.71	−2.4 (−7.9, +3.1)	0.86
pMg	+0.7 (−1.3, +2.7)	0.92	−0.6 (−2.6, +1.4)	0.95	−1.9 (−4.1, +0.3)	0.36	−1.6 (−4.3, +1.1)	0.74
pCr	+2.6 (−1.3, +6.5)	0.65	+5.0 (+1.1, +8.9)	0.11	+7.2 (+2.7, +11.7)	0.02^b^	+1.5 (−4.4, +7.4)	0.97
Urine chemistry and renal function								
eGFR	−1.9 (−4.1, +0.3)	0.39	−4.2 (−6.4, −2.0)	0.002^b^	−4.3 (−6.7, −1.9)	0.005^b^	−4.1 (−7.2, −1.0)	0.09
TmCa/GFR	+0.7 (−5.4, +6.8)	0.99	+0.4 (−5.7, +6.5)	0.99	+4.2 (−2.5, +10.9)	0.68	−2.6 (−11.4, +6.2)	0.96
TmP/GFR	−13.6 (−18.3, −8.9)	<0.0001^b^	−6.2 (−10.9, −1.5)	0.08^b^	−7.6 (−12.9, −2.3)	0.04^b^	−5.4 (−12.5, +1.7)	0.51^b^
2 h uCa/Cr	−14.7 (−45.1, +15.7)	0.82	−37.0 (−67.4, −6.6)	0.13^b^	−28.4 (−62.1, +5.3)	0.43	−4.8 (−49.9, +40.3)	0.99
2 h uP/Cr	+4.5 (−10.6, +19.6)	0.95	+9.3 (−6.0, +24.6)	0.70	+23.8 (+6.9, +40.7)	0.06^b^	+15.7 (−6.8, +38.2)	0.60^b^
2 h uMg/Cr	−6.1 (−19.8, +7.6)	0.86	+10.7 (−3.0, +24.4)	0.51	+22.6 (+7.1, +38.1)	0.04^b^	+21.5 (+0.9, +42.1)	0.24

*Note*: Values are mean percentage differences between the groups (%Diff) and 95% confidence intervals (95% CIs) for comparison of women with HIV initiated on TDF‐based antiretroviral therapy in pregnancy (WWH) versus women without HIV (REF). Results were obtained from Scheffé post hoc tests for group× timepoint interaction terms in four‐time‐point hierarchical repeated‐measures ANOVA models, which included participant ID (nested by group), group, timepoint, and group × timepoint interaction. Variables were transformed into natural logarithms and multiplied by 100 before data analysis. A plus symbol for differences shows that WWH had higher values, and a minus sign shows that WWH had lower values compared to REF.

Abbreviations: 1,25(OH)_2_D = 1,25‐dihydroxyvitamin D (pmol/L); 2 h uCa/Cr = 2 h fasting ratio of urinary calcium to creatinine 100×mmol/mmol; 2 h uMg/Cr = 2 h fasting ratio of urinary magnesium to creatinine (mmol/mmol); 2 h uP/Cr = 2 h fasting ratio of urinary phosphorus to creatinine (mmol/mmol); 25OHD = 25‐hydroxyvitamin D (nmol/L); BALP = bone‐specific alkaline phosphatase (μg/L); CTX = C‐terminal telopeptide (ng/L); eGFR = estimated glomerular filtration rate (CKD, ml/min/1.73 m^2^); FGF23 = intact fibroblast growth factor‐23 (ng/L); L14, L26 = 14, 26 weeks lactation, respectively; NPNL = at least 3 months after lactation when neither pregnant nor lactating; P1NP = procollagen type 1 N‐terminal propeptide (μg/L); P1NP/CTX = ratio of P1NP to CTX (1000×μg/ng); P36 = 36 weeks of pregnancy; pAlb = plasma albumin (g/L); pCa = plasma calcium (mmol/L); pCacorr = albumin‐corrected plasma calcium (Payne, mmol/L); pCr = plasma creatinine (μmol/L); pMg = plasma magnesium (mmol/L); pP = plasma phosphate (mmol/L); PTH = parathyroid hormone (ng/L); TALP = total alkaline phosphatase U/L; TmCa/GFR = renal tubular maximum reabsorption of calcium per unit volume of glomerular filtrate (mmol/L); TmP/GFR = renal tubular maximum reabsorption of phosphate per unit volume of glomerular filtrate (mmol/L).

^a^ The number of data points per analyte by group and timepoint are given in Supplement 3 in Data S1.

^b^ Significant group difference in cross‐sectional models with adjustment for predictors (mean percentage difference [95% CI] and *p* values from these models are in Supplement 4 in Data S1).

### Longitudinal biochemical changes in REF

In REF, the majority of measures increased from P36 to lactation except for TALP, 1,25(OH)_2_D, 25OHD, pCa_corr_, eGFR, uCa/Cr, and uP/Cr, which decreased (Figs. 2 and 3, Table 3). From L14 to L26, BALP, 25OHD, and eGFR increased, pCa, pCa_corr_, pP, and pCr decreased, with no significant changes in other analytes (Table 3). Between L26 and NPNL, all bone turnover markers, FGF23, and eGFR decreased, while pCa, pCa_corr_, pMg, pCr, and TmCa/GFR increased (Table 4). In general, values at NPNL were higher than or similar to those at P36 (Table 4), except for CTX, TALP, 1,25(OH)_2_D, eGFR, uCa/Cr, and uP/Cr, which were lower. The P1NP/CTX ratio increased from pregnancy into lactation (Table 3) and from lactation to NPNL (Table 4), indicating a shift in the balance between bone resorption and bone formation. The BALP/TALP ratio (100 × μg/L per U/L) in REF was (geometric mean [25th, 75th percentile]): 12.8 (9.9, 16.4) at P36, 29.3 (26.0, 32.9) at L14, 33.2 (29.8, 39.2) at L26, and 27.9 (23.4, 35.5) at NPNL. The ratio was lower at P36 compared to other timepoints, likely due to the contribution of placental alkaline phosphatase, and highest at L26.

Overall, the biochemical profile in REF at P36, when judged against values at NPNL, was for raised CTX, TALP, 1,25(OH)_2_D, eGFR, uCa/Cr, and uP/Cr, with lowered P1NP, BALP, PTH, FGF23, pAlb, pCa, pMg, pCr, and TmCa/GFR (Fig. 4*A*). In lactation, the profile was for raised bone resorption and formation markers, FGF23, pP, TmP/GFR and lowered pCa, pMg, pCr, TmCa/GFR.

### Biochemical differences between WWH and REF

In general, the patterns of change in WWH across the four timepoints were similar to those in REF (Figs. 2 and 3). However, within these overall patterns, there were significant group differences for some analytes, either in the value at one or more timepoints (Fig. 4 *B*, Table 5, Supplement 4 in Data S1) or in change between timepoints (Tables 3 and 4), giving rise to significant group × timepoint interactions in the four‐, three‐, and/or two‐timepoint models (Supplement 5 in Data S1). For most analytes with significant group × timepoint interactions, the effects were generally related to differences between WWH and REF in the magnitude of change between P36 and later timepoints, with fewer group differences in change across time during and after lactation (Supplement 5 in Data S1).

Overall WWH had higher PTH than REF and lower 1,25(OH)_2_D and TmP/GFR across all four timepoints, with no evidence of group × timepoint interactions (overall mean %Diff [95% CI] WWH‐REF: PTH +31.4 [+19.8, +42.9]% *p <* 0.0001; 1,25(OH)_2_D −9.3 [−14.4, −4.2] *p =* 0.0004; TmP/GFR −8.8 [−13.5, −4.1]% *p =* 0.0003).

WWH had lower pP at P36 (mean %Diff [95% CI]: −10.1 [−13.8, −6.4]%, *p <* 0.0001) than at later timepoints (−3.0 [−7.3, −1.3]%, *p* = 0.17), resulting in significant group × timepoint interactions in models including P36 (Supplement 5 in Data S1). P1NP was also lower in WWH than REF at P36, but higher at NPNL and not significantly different during lactation (Fig. 2, Table 5). Consequently, the P1NP/CTX ratio was lower in WWH at P36, less so in lactation, and not significantly different at NPNL (Fig. 2, Table 5). The ratio of BALP/TALP was not significantly different between the groups at any timepoint.

Conversely, for many analytes, group differences emerged only after pregnancy, generally demonstrated by significant group × timepoint interactions in models involving P36, but not in those with P36 excluded (Supplement 5 in Data S1). WWH had higher CTX, BALP, TALP, pCr, and uMg/Cr and lower eGFR during and after lactation (Fig. 4*B*). For these analytes, the overall mean %Diff (95% CI) between WWH and REF in three‐timepoint models without P36 were as follows: CTX +14.8 (+1.1, +28.5)% *p =* 0.04; BALP +18.8 (+7.4, +30.2)% *p =* 0.001; TALP +17.8 (+8.6, +27.0)% *p =* 0.0002; pCr +4.4 (−0.5, +9.3)% *p =* 0.09; uMg/Cr +15.9 (+1.8, +30.0)% *p =* 0.03; eGFR −3.9 (−7.6, −0.2)% *p =* 0.04. Notably pCa was lower in WWH at L14 (−5.3 (−6.5, −4.1)% *p <* 0.0001), reflected in significant group × timepoint interactions in models including L14 (Supplement 5 in Data S1). Additionally, WWH had higher 25OHD than REF at L14 but, unlike REF, had no increase in 25OHD between L14 and L26 and a downward trend from P36 to NPNL (Tables 2, 3, 4 and Supplement 4 in Data S1). This difference in longitudinal pattern for 25OHD was reflected in significant group × timepoint interactions (Supplement 5 in Data S1).

Several of the variables investigated as potential confounders of differences between WWH and REF were found to be predictive of specific biochemical analytes at one or more timepoints (Supplement 4 in Data S1). In many of these adjusted models, group differences increased in statistical significance (compare with Table 2), but in most cases, the magnitudes of the differences were only marginally affected (compare with Table 4). There was no evidence of any influence of these variables on group differences in change between timepoints.

## Discussion

This extensive, longitudinal study of biochemical markers of bone and calcium metabolism in Ugandan women was conducted in order to investigate the mechanisms underlying observed postpartum differences in aBMD^(^ ^11^ ^)^ and breast‐milk calcium^(^ ^18^ ^)^ between women with HIV recently initiated onto ART and those without HIV. These women were urban residents receiving antenatal care in a public hospital and as such were likely to be resource‐limited with a marginal diet and low calcium intake (<400 mg/day), in common with participants of other studies in the same population (e.g., Kiwanuka et al., Cormick et al.^(^ ^28^, ^29^ ^)^).

In both groups of women, we observed a consistent pattern of changes in markers of bone turnover and mineral metabolism from late pregnancy into lactation and 3–6 months after lactation, resembling in many respects those reported in Western countries and West Africa.^(^ ^13^, ^14^, ^30^, ^31^ ^)^ Similarly to other studies, the key characteristics of late pregnancy were as follows: elevated CTX (bone resorption marker) and 1,25(OH)_2_D, lower bone formation markers (P1NP, BALP), PTH, FGF23, and TmCa/GFR relative to that after lactation. In lactation, all bone resorption and formation markers were elevated, while 1,25(OH)_2_D, PTH, and FGF23 returned to concentrations close to those after lactation. There was also greater renal reabsorption of calcium and phosphorus postpartum, as shown by increases in TmCa/GFR and TmP/GFR, and decreases in 2 h fasting urinary outputs of these minerals. By 3–6 months after lactation when the women were neither pregnant nor lactating, although both CTX and P1NP had decreased from the high concentrations in lactation, the P1NP/CTX ratio was at its highest level, suggesting a shift in the balance of bone turnover toward bone formation. In addition, renal calcium reabsorption was further elevated after lactation, as shown by an additional increase in TmCa/GFR.

Within this general pattern, there were significant differences between WWH and REF. CTX and BALP were consistently higher in WWH during and after lactation, while P1NP was lower in pregnancy but higher after lactation. As a result, the P1NP/CTX ratio was lower in WWH (favoring bone resorption) in both pregnancy and lactation, normalizing after lactation. PTH was higher and TmP/GFR was lower in WWH throughout, and 2 h uP/Cr was higher and eGFR was lower in lactation and after lactation. These findings are in line with previous reports in adults of impaired renal function and altered phosphate metabolism associated with TDF.^(^ ^8^, ^9^, ^10^ ^)^ Notably 1,25(OH)_2_D was lower in WWH throughout, despite adequate 25OHD status, possibly also due to renal toxic effects of TDF. Although there was no evidence of a difference in TmCa/GFR, WWH had significantly lower pCa and 2 h uCa/Cr at 14 weeks of lactation, suggesting greater metabolic stress in providing sufficient calcium for breast‐milk production and bone mineralization.

To the best of our knowledge, this is the first study to describe changes in bone turnover markers, calciotropic hormones, and other aspects of bone mineral biochemistry in a longitudinal cohort of pregnant and lactating African WWH on TDF‐based triple ART compared to women without HIV. Studies conducted in men and nonpregnant, nonlactating women have reported early increases in bone resorption markers and delayed increases in bone formation markers after initiation of ART, and this is thought to create a catabolic window for bone mineral loss.^(^ ^9^, ^32^ ^)^ Greater bone mineral loss and changes in bone turnover markers have been observed with TDF‐based ART compared to other regimens.^(^ ^9^ ^)^ In our study, WWH, who had been initiated on TDF‐based ART earlier in pregnancy, had lower P1NP at P36, higher CTX and BALP during lactation, and higher P1NP and BALP after lactation than REF. These data are, therefore, consistent with the hypothesis that TDF‐associated bone demineralization is related to increased bone turnover^(^ ^9^, ^32^ ^)^ and decreased bone formation.^(^ ^33^ ^)^

Our study also revealed elevated PTH, lower 1,25(OH)_2_D, and alterations in calcium‐phosphate‐magnesium metabolism independent of 25OHD in WWH, in line with previous reports of effects of TDF‐based ART.^(^ ^4^, ^34^, ^35^, ^36^ ^)^ The low concentrations of many of the markers in pregnancy compared to after lactation could be partly ascribed to hemodilution. However, there was no evidence of any consistent differences between WWH and REF in pAlb or in the BALP/TALP ratio, indicating that it was unlikely that either hemodilution or liver involvement was responsible for the biochemical differences observed between the groups.

In our study, WWH had greater decreases in aBMD during lactation, with only partial skeletal recovery at 3–6 months after lactation,^(^ ^11^ ^)^ and they had higher breast‐milk calcium concentrations in the first months of lactation.^(^ ^18^ ^)^ The biochemical differences we observed suggest that TDF was responsible for these disparities. The physiological mechanisms that produce TDF effects are not clear, and many hypotheses have been proposed to explain them. Suggestions include TDF‐induced mitrochondrial toxicity and DNA‐synthesis inhibition, perturbations in proximal renal tubular function, and alterations in vitamin D metabolism.^(^ ^8^, ^10^ ^)^ Studies have consistently described an increase in PTH following TDF initiation,^(^ ^34^, ^35^, ^36^ ^)^ associated with loss of bone mineral. A potential mechanism for this is a direct inhibitory effect of TDF on calcium‐sensing receptors (CaSR) in the parathyroids, resulting in raised PTH.^(^ ^37^ ^)^ This has not been demonstrated in human studies; however, mutations in the CaSR are recognized causes of hyperparathyroidism. In addition, mammary gland CaSR are involved in the regulation of calcium transport to milk and PTH‐related protein (PTHrP) production.^(^ ^14^, ^18^ ^)^ PTHrP plays an active role in calcium homeorhesis during pregnancy and lactation,^(^ ^13^, ^14^ ^)^ and a higher plasma PTHrP is associated with greater bone loss in early lactation^(^ ^13^ ^)^ and with hypophosphatemia in those with HIV on TDF.^(^ ^38^ ^)^ Therefore, it is plausible that TDF‐based ART in WWH may have altered parathyroid and mammary gland CaSR and affected PTH/PTHrP activity in such a way as to lead to the observed effects on aBMD, breast‐milk calcium, bone turnover, and mineral metabolism. In addition, the lower 1,25(OH)_2_D in WWH may have resulted in reduced intestinal calcium absorption. For women on a low‐calcium diet, this could limit calcium supply. This, combined with the higher breast‐milk calcium content, may account for the apparent increase in calcium metabolic stress in WWH implied by lower pCa and 2 h uCa/Cr at 14 weeks of lactation, despite an increased bone mineral mobilization.

Our study has several strengths. We collected extensive data on bone turnover markers, calciotropic hormones, and renal mineral handling in the same individuals across pregnancy, lactation, and the postlactation period. The study included a reference group of women without HIV, which permitted direct comparison of biochemical measures in WWH with reference data collected in the same community and analyzed in the same laboratory with the same assays. All the participants with HIV were previously ART‐naïve, initiated a single ART regimen (TDF/3TC/EFV), and reported good adherence to ART. In addition, the participants breastfed for at least 6 months, providing an opportunity to look at the additive effect of HIV/ART and lactation on bone and mineral biomarkers. The study is limited by the lack of measurements before ART was started and by the lack of data on HIV viral load in WWH. Plasma tenofovir concentrations were not measured, so a dose–response effect could not be explored. In addition, there were no prepregnancy measures, and values obtained at 3–6 after lactation may not fully represent the nonpregnant, nonlactating state if some degree of homeorhesis was still occurring. The representativeness of the reference group may be limited by the reduced number available for follow‐up at 3–6 months after lactation. Finally, the study could not separate the effects of HIV and ART, and the results may not be generalizable to WWH on non‐TDF‐based ART, those in high‐income settings, or to pregnant and lactating women on pre‐exposure ART prophylaxis (PrEP) or treatment for hepatitis B.

In conclusion, resource‐limited African women in Uganda experienced changes in biochemical markers of bone and mineral metabolism during pregnancy and lactation similar to those experienced by women in other settings. However, those women with HIV who had initiated TDF‐containing triple ART earlier in pregnancy had higher bone turnover, elevated PTH, lower 1,25(OH)_2_D, and disturbances in calcium and phosphorus metabolism during and after lactation, which may lie behind their greater lactational bone mineral mobilization and higher breast‐milk calcium.^(^ ^11^, ^18^ ^)^ Some of these endocrine changes resemble those reported in association with TDF‐based ART in men and nonpregnant women. The possible use of vitamin D and calcium to lower PTH and increase bone mineral density has been suggested, and some short‐term positive effects of vitamin D therapy with and without calcium supplementation have been demonstrated.^(^ ^39^, ^40^ ^)^ However, the benefits of vitamin D supplementation in individuals with good vitamin D status are unknown, and unexpected long‐term effects of calcium supplementation in African women and children have been reported.^(^ ^41^ ^)^ Thus, further studies are needed to explore mechanisms in more detail and to investigate whether the use of TDF‐based ART by African women during pregnancy and lactation has long‐term consequences for the bone health of the mother and for the growth of the child. In particular, clinical endpoint data will be required to determine the implications of the findings of this study and to guide consideration of potential interventions.

## Disclosure

All authors state that they have no conflicts of interest.

## Author Contributions

**Florence Nabwire:** Conceptualization; data curation; formal analysis; funding acquisition; investigation; methodology; supervision; writing – original draft; writing – review and editing. **Matthew M. Hamill:** Conceptualization; writing – original draft; writing – review and editing; methodology. **Mary Glenn Fowler:** Conceptualization; methodology; supervision; writing – review and editing. **Josaphat Byamugisha:** Conceptualization; methodology; supervision; writing – review and editing. **Adeodata Kekitiinwa:** Conceptualization; methodology; writing – review and editing. **Ann Prentice:** Conceptualization; data curation; formal analysis; funding acquisition; methodology; supervision; writing – original draft; writing – review and editing.

### Peer Review

The peer review history for this article is available at https://www.webofscience.com/api/gateway/wos/peer-review/10.1002/jbmr.4866.

## Supporting information


**Data S1.** Supporting Information.

## Data Availability

Data from the Gumba Study are archived with UKRI‐MRC, London, UK. The procedure for requesting data access can be found at https://www.ukri.org/publications/access-to-data-from-the-mrc-science-archive-application-form/.
