# Supplementary material for: Biochemical Markers of Calcium and Bone Metabolism during and after Lactation in Ugandan Women with HIV on Universal Maternal Antiretroviral Therapy
Source: J Bone Miner Res. 2023 Jul 24;38(9):1296–311. doi: 10.1002/jbmr.4866 (PMC10947145; doi:10.1002/jbmr.4866)
Supplement: Supplementary file 1 — Data S1. Supporting Information. [file JBMR-38-1296-s001.docx]

SUPPLEMENTARY MATERIAL

## Supplement 1: Collection, processing and analysis of biological samples

Blood was obtained by venepuncture and processed as serum, lithium heparin plasma and EDTA plasma. Blood tubes were centrifuged at 3000 rpm at 4^0^C within two-hours of collection and aliquots prepared using disposable pipettes. The total volume of urine produced over the two hours was thoroughly mixed, and processed within three hours of collection. Urine aliquots (5ml) were acidified with 1 drop (50µl) each of 37% concentrated hydrochloric acid (Sigma: 12 Molar, Specific gravity 1.18). A second 5ml urine aliquot was stored unacidified. Samples were stored at -80^o^C prior to analysis. 1,25(OH)_2_D and intact FGF23 were assayed at the Nutritional Biomarker Laboratory of the MRC Epidemiology Unit, Cambridge, UK. All other assays were performed at the MRC Elsie Widdowson Laboratory (MRC EWL), Cambridge, UK.

Singleton analyses using IDS iSyS platform and kits (Immunodiagnostics Systems Ltd, Tyne and Wear, UK) were used to measure β-CTX in LH plasma, intact PTH and P1NP in serum. Plasma BALP was quantified in LH plasma using the Microvue BALP ELISA assay that has low cross-reactivity with liver alkaline phosphatase (Quidel Corporation, Athens, USA). Serum intact FGF23 and 1,25(OH)_2_D were measured in duplicate by ELISA. Serum 25-hydroxyvitamin D (25(OH)D) was analysed in duplicate using the DiaSorin chemiluminescent immunoassay (Liaison; DiaSorin Inc., Stillwater, MN, USA). At the time of these measurements, the laboratory at MRC EWL participated in both the international Vitamin D External Quality Assessment Scheme (DEQAS, www.deqas.org) and held a certificate of proficiency for 25OHD. Additionally the laboratory participated in the Vitamin D Standardization Program (VDSP, (https://ods.od.nih.gov/Research/vdsp.aspx)) which standardised its performance of the Diasorin Liaison assay to internationally accepted reference methods at National Institute of Standards and Technology (www.nist.gov) and the University of Ghent. Quality assurance for PTH assays was performed as part of the National External Quality Assessment Scheme (www.ukneqas.org.uk). For the other analytes, standards and reference materials supplied by the manufacturers were used for quality assurance. In addition, for intact FGF23, an in-house serum quality control was included with each batch to monitor precision at the lower end of the assay because the majority of samples had values between the low kit standards (STD 1 (0 ng/L) and STD 2 (22 ng/L)).

All other plasma and urine assays were performed in duplicate in LH plasma and acidified urine, respectively, on a Konelab analyser (Thermo Fischer Scientific, Vantaa, Finland). The manufacturer’s kits for albumin (bromocresol purple), calcium (arsenazo III), phosphate (ammonium molybdate), magnesium (xylidyl blue 1), TALP (IFCC plus at 37 ^0^C) and creatinine (Cr) (Jaffé) were used. Measured plasma creatinine (pCr) values were adjusted to be traceable to the international reference standard by the manufacturer’s formula: creatinine compensated µmol/L = (measured result -26.8) µmol/L x 1.168. Fourteen urine samples (7 P36 and 34 L14) had a calcium concentration below the detection limit and were assigned a nominal value of 0.01 mmol/L (half the detection limit of 0.02 mmol/L) allowing these data points to contribute to the analysis. Low, medium, and high control samples and materials supplied by kit manufacturers (Roche serum control (Roche Diagnostic Corporation, Indianapolis, USA), Lyphochek (Bio-Rad Laboratories, Herts, UK), NEQAS Clin Chem (Birmingham, UK)) and internal plasma drift controls were used for quality assurance.

Plasma calcium was normalised for albumin using the Payne equation:^(1)^ albumin corrected plasma Ca (pCa_corr_, mmol/L) = measured plasma Ca, (pCa, mmol/L) + ([40 - plasma albumin (pAlb, g/L)]*0.02). The ratio of pAlb at NPNL to pAlb in pregnancy was used as a multiplication factor to consider possible effects of haemodilution in pregnancy on blood concentrations, with pAlb at NPNL representing pAlb pre-pregnancy.^(2)^Estimated glomerular filtration rate (eGFR) based on plasma creatinine concentrations (pCr) was calculated using the Chronic Kidney Disease – Epidemiology Collaboration (CKD-EPI) equation for females,^(3)^ without the correction for black ethnicity consistent with previous studies in African populations.^(4,5)^ For pCr ≤ 62µmol/L (0.7mg/dL): eGFR (mL/min/1.73m^2^) = 144 × ((pCr, µmol/L*0.01131222)/0.7)^-0.329^ × (0.993)^age^. For pCr > 62µmol/L(0.7mg/dL): eGFR (mL/min/1.73m2) = 144 × ((pCr, µmol/L*0.01131222)/0.7)^-1.209^ × (0.993)^age^. Renal tubular maximum per unit volume of glomerular filtrate, for reabsorption of phosphate (TmP/GFR) and calcium (TmCa/GFR) were calculated as follows: TmP/GFR (mmol/L) = (a) TRP×pP if TRP ≤0.86 and (b) α×pP if TRP >0.86, where TRP = (1−(( uP/pP)×(pCr/uCr))) and α = ((0.3×TRP) / (1−(0.8× TRP))).^(6)^ TmCa/GFR (mmol/L) = ((0.56×pCa) − (uCa/uCr)×pCr) / (1 − 0.08 log_e_ (0.56×pCa /(uCa/uCr)×pCr).^(7)^

**References**

1. Tietz NW, Finley PR, Pruden E, Amerson AB. Clinical Guide to Laboratory Tests. 2nd Edition ed. Tietz NW, editor. Philadelphia: Saunders; 1990. 931 pp.

2. Kaur M, Godber IM, Lawson N, Baker PN, Pearson D, Hosking DJ. Changes in serum markers of bone turnover during normal pregnancy. Ann Clin Biochem. 2003;40:508-13.

3. Levey AS, Stevens LA, Schmid CH, et al. A new equation to estimate glomerular filtration rate. Ann Intern Med. 2009;150:604-12.

4. van Deventer HE, George JA, Paiker JE, Becker PJ, Katz I. Estimating glomerular filtration rate in black South Africans by use of the modification of diet in renal disease and Cockcroft-Gault equations. Clin Chem. 2008;54:1197-202.

5. Hamill MM, Pettifor JM, Ward KA, Norris SA, Prentice A. Changes in bone mineral density, body composition, vitamin D status, and mineral metabolism in urban HIV-positive South African women over 12 months. J Bone Miner Res. 2017;32:1615-24.

6. Payne RB. Renal tubular reabsorption of phosphate (TmP/GFR): indications and interpretation. Ann Clin Biochem. 1998;35:201-16.

7. Nordin BEC, Horsman A, Aaron J. Diagnostic Procedures. Edinburgh: Churchill Livingstone; 1976.

## Supplement 2: Flow of participants in the biochemical component of the study

## Supplement 3: Summary of analytes and numbers included in final hierarchical models by group and timepoint.

|  | P36 | |  |  | L14 | |  | L26 | |  | NPNL | |  |
| --- | --- | --- | --- | --- | --- | --- | --- | --- | --- | --- | --- | --- | --- |
|  | WWH (*n* = 83) | REF (*n* = 81) |  |  | WWH *(n* = 83) | REF *(n* = 81) |  | WWH *(n* = 69) | REF *(n* = 72) |  | WWH *(n* = 66) | REF (*n* = 32) |  |
| Bone turnover markers | |  |  |  |  |  |  |  |  |  |  |  |  |
| CTX | 83 | 80 |  |  | 81 | 81 |  | 69 | 72 |  | 66 | 32 |  |
| P1NP | 83 | 81 |  |  | 81 | 81 |  | 69 | 72 |  | 66 | 32 |  |
| BALP | 83 | 81 |  |  | 81 | 81 |  | 69 | 71 |  | 64 | 32 |  |
| TALP | 83 | 81 |  |  | 83 | 81 |  | 69 | 72 |  | 65 | 32 |  |
| P1NP/CTX | 83 | 80 |  |  | 81 | 81 |  | 69 | 72 |  | 66 | 31 |  |
| Hormones and vitamin D status | | |  |  |  |  |  |  |  |  |  |  |  |
| PTH | 83 | 81 |  |  | 81 | 81 |  | 69 | 72 |  | 66 | 32 |  |
| FGF23 | 82 | 80 |  |  | 80 | 81 |  | 68 | 72 |  | 66 | 32 |  |
| 1,25(OH)_2_D) | 82 | 80 |  |  | 83 | 81 |  | 69 | 72 |  | 66 | 31 |  |
| 25OHD | 83 | 81 |  |  | 82 | 81 |  | 69 | 72 |  | 66 | 32 |  |
| Plasma chemistry | |  |  |  |  |  |  |  |  |  |  |  |  |
| pAlb | 83 | 81 |  |  | 82 | 81 |  | 68 | 72 |  | 66 | 32 |  |
| pCa | 83 | 81 |  |  | 81 | 79 |  | 68 | 72 |  | 66 | 32 |  |
| pCa_corr_ | 83 | 81 |  |  | 81 | 79 |  | 68 | 72 |  | 66 | 32 |  |
| pP | 83 | 81 |  |  | 83 | 81 |  | 69 | 72 |  | 66 | 32 |  |
| pMg | 82 | 81 |  |  | 83 | 80 |  | 68 | 72 |  | 66 | 32 |  |
| pCr | 83 | 81 |  |  | 83 | 81 |  | 69 | 72 |  | 66 | 32 |  |
| Urine chemistry and renal function | | |  |  |  |  |  |  |  |  |  |  |  |
| eGFR | 82 | 81 |  |  | 82 | 81 |  | 68 | 72 |  | 66 | 32 |  |
| TmCa/GFR | 82 | 81 |  |  | 82 | 80 |  | 68 | 71 |  | 65 | 32 |  |
| TmP/GFR | 82 | 81 |  |  | 82 | 81 |  | 69 | 71 |  | 65 | 32 |  |
| 2h uCa/Cr | 82 | 81 |  |  | 82 | 81 |  | 69 | 71 |  | 65 | 32 |  |
| 2h uP/Cr | 82 | 81 |  |  | 82 | 80 |  | 68 | 71 |  | 64 | 32 |  |
| 2h uMg/Cr | 82 | 81 |  |  | 82 | 81 |  | 69 | 70 |  | 65 | 32 |  |

Values are number of samples analysed for each analyte; missing data points were due to insufficient or no sample. P36 = 36 weeks of pregnancy; L14, L26 = 14, 26 weeks lactation respectively; NPNL = at least 3 months post-lactation when neither pregnant nor lactating; WWH = women with HIV initiated on tenofovir-based ART during pregnancy (previously ART-naïve) who provided blood samples at 2 or more timepoints; REF = women without HIV who provided blood samples at 2 or more timepoints; ART = antiretroviral therapy; P36 = 36 weeks of pregnancy; L14, L26 = 14, 26 weeks lactation respectively; WWH = women with HIV initiated on tenofovir-based ART during pregnancy (previously ART-naïve) who provided blood samples at 2 or more timepoints; REF = women without HIV who provided blood samples at 2 or more timepoints; ART = antiretroviral therapy; CTX = C-terminal telopeptide (ng/L); P1NP = procollagen type 1 N-terminal-propeptide (µg/L); BALP = bone-specific alkaline phosphatase (µg/L); TALP = total alkaline phosphatase U/L; P1NP/CTX = ratio of P1NP to CTX (1000*µg/ng); PTH = parathyroid hormone (ng/L); FGF23 = intact fibroblast growth factor-23 (ng/L); 1,25(OH)_2_D = 1,25-dihydroxyvitamin D (pmol/L); 25OHD = 25-hydroxyvitamin D (nmol/L); pAlb = plasma albumin (g/L); pCa = plasma calcium (mmol/L); pCa_corr_ = albumin corrected plasma calcium (Payne, mmol/L); pP = plasma phosphate (mmol/L); pMg =plasma magnesium (mmol/L); pCr= plasma creatinine (µmol/L); eGFR = estimated glomerular filtration rate (CKD, ml/min/1.73m^2^); TmCa/GFR = renal tubular maximum reabsorption of calcium per unit volume of glomerular filtrate (mmol/L); TmP/GFR = renal tubular maximum reabsorption of phosphate per unit volume of glomerular filtrate (mmol/L); 2h uCa/Cr = 2h fasting ratio of urinary calcium to creatinine 100*mmol/mmol; 2h uP/Cr = 2h fasting ratio of urinary phosphorus to creatinine (mmol/mmol); 2h uMg/Cr = 2h fasting ratio of urinary magnesium to creatinine (mmol/mmol).

**Supplement 4.** Summary of percentage differences between WWH and REF at each timepoint in cross-sectional models with adjustment for independent predictors^a^.

|  | P36 | |  | L14 | |  | L26 | |  | NPNL | |
| --- | --- | --- | --- | --- | --- | --- | --- | --- | --- | --- | --- |
|  | % Diff (95% CI) | *p* |  | % Diff (95% CI) | *p* |  | % Diff (95% CI) | *p* |  | % Diff (95% CI) | *p* |
| Bone turnover markers | |  |  |  |  |  |  |  |  |  |  |
| CTX | -2.4 (-15.7, +10.9) | .72 ^pG+,pD-^ |  | +17.4 (+3.9, +30.9) | .01 ^Ag-,pD-,Ms-,Wt-^ |  | +17.1 (+1.8, +33.06 | .03 ^Ag-,pD-,Ms-,Wt-^ |  | +8.5 (-16.6, +33.6) | .51 ^Ag-,Wt-^ |
| P1NP | -22.0 (-36.1, -7.9) | .003^pG+^ |  | +3.2 (-7.0, +13.4) | .54 ^Ag-,pD-^ |  | -2.6 (-16.3, +11.1) | .70 ^Ag-,pD-,Wt-^ |  | +25.6 (+3.6, +47.6) | .02 ^Ag-,pD-,Wt-^ |
| BALP | +9.5 (-1.9, +20.9) | .10 ^pG+^ |  | +14.8 (+4.2, +25.4) | .007 ^Ht-^ |  | +19.9 (+7.0, +32.8) | .003 ^Ag-, Ms-^ |  | +29.5 (+10.5, +48.5) | .003 |
| TALP | -3.9 (-14.9, +7.1) | .49 ^Ht-^ |  | +19.1 (+11.3, +26.9) | <.0001 ^Ms-, Sx+^ |  | +12.9 (+1.9, +23.9) | .02 |  | +26.4 (+12.7, +40.1) | .0003 |
| P1NP/CTX | -21.2 (-34.9, -7.5) | .003 |  | -14.7 (-27.2, -2.2) | .02 |  | -18.9 (-32.8, -5.0) | .009 |  | +6.2 (-14.2, +26.6) | .55 |
| Hormones and vitamin D status | |  |  |  |  |  |  |  |  |  |  |
| PTH | +34.1 (+19.0, +49.2) | <.0001 |  | +33.4 (+18.3, +48.5) | <.0001 ^EB+, Wt+^ |  | +29.7 (+13.8, +45.6) | .0003 ^Wt+, Ht-^ |  | +31.1 (+14.0, +48.2) | .0006 ^Ag+^ |
| FGF23 | +15.7 (-4.3, +35.7) | .13 |  | -16.0 (-32.3, +0.3) | .06 ^Wt-^ |  | -19.8 (-36.6, -3.2) | .02 |  | +7.3 (-16.2, +30.8) | .54 |
| 1,25(OH)_2_D | -12.3 (-19.6, -5.0) | .001 ^Wt+, Ht-^ |  | -4.7 (-16.5, +7.1) | .43 ^cD+, Ht-^ |  | -5.5 (-12.0, +1.0) | .10 ^Sx-^ |  | -12.2 (-23.6, -0.8) | .04 ^Ht-^ |
| 25OHD | +4.0 (-7.2, +15.2) | .48 |  | +10.8 (+0.1, +21.5) | .05 |  | -6.4 (-17.6, +4.8) | .26 |  | -16.4 (-31.5, -1.3) | .04 ^pD+^ |
| Plasma chemistry | |  |  |  |  |  |  |  |  |  |  |
| pAlb | -0.3 (-3.0, +2.4) | .81 ^Ag-,Ht+^ |  | -2.7 (-5.1, -0.3) | .02 |  | +1.2 (-1.5, +3.9) | .41 ^Ms+, Wt-, Ht+^ |  | -2.2 (-5.1, +0.7) | .15 ^pG+^ |
| pCa | -1.2 (-2.4, 0.0) | .05 ^pG+^ |  | -4.9 (-6.5, -3.3) | <.0001 ^EB-^ |  | +0.6 (-1.2, +2.4) | .50 ^Ht+^ |  | -1.7 (-3.3, -0.1) | .04 ^Ag-, Sx-^ |
| pCa_corr_ | -1.2 (-2.2, -0.2) | .02 |  | -4.3 (-5.7, -2.9) | <.0001 |  | +0.3 (-1.1, +1.7) | .72 ^Ht+^ |  | -1.2 (-2.8, +0.4) | .14 ^Ag-^ |
| pP | -9.9 (-14.4, -5.4) | <.0001 |  | -2.9 (-7.4, +1.6) | .21 ^Ag-^ |  | -2.2 (-6.9, +2.5) | .37 ^Ag-^ |  | -2.4 (-7.9, +3.1) | .53 |
| pMg | +0.6 (-1.8, +3.0) | .65 |  | -0.6 (-2.8, +1.6) | .56 ^Ag+^ |  | -1.9 (-4.4, +0.6) | .14 ^Ag+, Ms+^ |  | -0.4 (-3.5, +2.7) | .79 |
| pCr | +2.5 (-4.0, +9.0) | .46 |  | +5.1 (-0.2, +10.4) | .07 |  | +7.7 (+2.0, +13.4) | .009 ^pD+^ |  | +3.1 (-2.4, +8.6) | .42 |
| Urine chemistry and renal function | |  |  |  |  |  |  |  |  |  |  |
| eGFR | -0.7 (-2.9, +1.5) | .53 ^Ag-^ |  | -3.9 (-7.4, -0.4) | .03 ^Ag-^ |  | -4.4 (-7.3, -1.5) | .005 ^Ag-^ |  | -4.6 (-10.5. +1.3) | .13 ^Ag-^ |
| TmCa/GFR | +0.1 (-7.0, +7.2)) | .70 ^pG+,pD+,Wt-^ |  | +0.1 (-7.3, +7.5) | .99 ^Ag-,pG-^ |  | +5.6 (-4.2, +15.4) | .27 ^Ag-,cD-,Sx-^ |  | -2.3 (-10.7, +6.1) | .60 |
| TmP/GFR | -13.6 (-19.7, -7.5)) | <.0001 |  | -6.0 (-11.3, -0.7) | .03 ^Wt+,Ht-^ |  | -8.3 (-14.0, -2.6) | .005 |  | -13.3 (-25.1, -1.5) | .03 ^Ag-,BD-^ |
| 2h uCa/Cr | -4.6 (-42.8, +33.6) | .81 ^pD-,Wt+^ |  | -38.5 (-76.1, -0.9) | .05 |  | -30.3 (-81.8, +21.2) | .25 ^cD+,Sx+^ |  | -8.1 (-48.7, +32.5) | .70 |
| 2h uP/Cr | +6.2 (-7.5, +19.9) | .38 ^pG+^ |  | +1.2 (-20.2, +22.6) | .91 ^EB+^ |  | +30.9 (+9.5, +52.3) | .005 ^pD-,Sx+^ |  | +28.2 (+1.3, +55.1) | .04 ^BD+, Sx+^ |
| 2h uMg/Cr | -7.4 (-22.9, +8.1) | .35 |  | +12.4 (-5.0, +29.8) | .17 ^pG+^ |  | +22.2 (+5.1, +39.3) | .01 ^pG+^ |  | +16.2 (-15.6, +48.0) | .14 |

Values are mean percent differences between the groups (%Diff) and 95% confidence intervals (95% CI) for comparison of women with HIV initiated onto TDF-based antiretroviral therapy in pregnancy (WWH) versus women without HIV (REF). A + symbol for differences shows that WWH had higher values and a - sign shows that WWH had lower values compared to REF. Superscript symbols denote positive (+) and negative (-) predictors (p≤.05) in parsimonious cross-sectional models with dependant variables transformed to natural logarithms and independent variables group (WWH/REF = 1/0) and the following potential predictors: at P36 these were Ag = maternal age (years), Ht = height (log_e_ transformed), Wt = body weight (log_e_ transformed), pG = primigravidity (1/0), pD = previous use of depot medroxyprogesterone acetate (DMPA) (1/0); at L14 and L26 these were as for P36 plus cD = current use of DMPA (1/0), EB = current exclusive breast-feeding (1/0), Ms = menses resumed (1/0), Sx = infant sex (M/F, 1/2); at NPNL same as P36 plus Sx = infant sex (M/F, 1/2), BD = breast-feeding duration (months).

P36 = 36 weeks of pregnancy; L14, L26 = 14, 26 weeks lactation respectively; NPNL = at least 3 months post-lactation when neither pregnant nor lactating; WWH = women with HIV initiated on tenofovir-based ART during pregnancy (previously ART-naïve) who provided blood samples at 2 or more timepoints; REF = women without HIV who provided blood samples at 2 or more timepoints; ART = antiretroviral therapy; CTX = C-terminal telopeptide (ng/L); P1NP = procollagen type 1 N-terminal-propeptide (µg/L); BALP = bone-specific alkaline phosphatase (µg/L); TALP = total alkaline phosphatase U/L; P1NP/CTX = ratio of P1NP to CTX (1000*µg/ng); PTH = parathyroid hormone (ng/L); FGF23 = intact fibroblast growth factor-23 (ng/L); 1,25(OH)_2_D = 1,25-dihydroxyvitamin D (pmol/L); 25OHD = 25-hydroxyvitamin D (nmol/L); pAlb = plasma albumin (g/L); pCa = plasma calcium (mmol/L); pCa_corr_ = albumin corrected plasma calcium (Payne, mmol/L); pP = plasma phosphate (mmol/L); pMg =plasma magnesium (mmol/L); pCr= plasma creatinine (µmol/L); eGFR = estimated glomerular filtration rate (CKD, ml/min/1.73m^2^); TmCa/GFR = renal tubular maximum reabsorption of calcium per unit volume of glomerular filtrate (mmol/L); TmP/GFR = renal tubular maximum reabsorption of phosphate per unit volume of glomerular filtrate (mmol/L); 2h uCa/Cr = 2h fasting ratio of urinary calcium to creatinine 100*mmol/mmol; 2h uP/Cr = 2h fasting ratio of urinary phosphorus to creatinine (mmol/mmol); 2h uMg/Cr = 2h fasting ratio of urinary magnesium to creatinine (mmol/mmol).

^a^ The number of data points per analyte by group and timepoint are given in Supplement 3.

**Supplement 5.** Significance of differences between WWH and REF in patterns of change between timepoints^a^.

|  | |  | | | |  | | |  | | | | |  | | |  | | | | |  | | | |  | | | |  |  |
| --- | --- | --- | --- | --- | --- | --- | --- | --- | --- | --- | --- | --- | --- | --- | --- | --- | --- | --- | --- | --- | --- | --- | --- | --- | --- | --- | --- | --- | --- | --- | --- |
|  | 4-timepoint  group*tpt  *p*-value  *P36-L14-L26-NPNL* | | | | | | 3-timepoint  group*tpt  *p*-value  *P36-L14-L26* | | | 3-timepoint group*tpt  *p*-value  *L14-L26-NPNL* | | | | | | 2-timepoint  group*tpt  *p*-value  *P36-L14* | | | 2-timepoint group*tpt  *p*-value  *L14-L26* | | | | | 2-timepoint group*tpt  *p*-value  *L26-NPNL* | | | 2-timepoint group*tpt  *p*-value  *P36-NPNL* | | | | |
| Bone turnover markers | | | |  | | | |  | | | |  |  | | | | |  | | |  | | | |  | | | |  |  |  |
| CTX | | | .06 | | .02 | | | | | | .97 | | | | .02 | | | | | .91 | | | .76 | | | | | .11 | | |  |
| P1NP | | | <.0001 | | .0001 | | | | | | .007 | | | | .0003 | | | | | .49 | | | .02 | | | | | .0004 | | |  |
| BALP | | | .003 | | .01 | | | | | | .26 | | | | .05 | | | | | .70 | | | .50 | | | | | .003 | | |  |
| TALP | | | <.0001 | | .0002 | | | | | | .07 | | | | .0002 | | | | | .22 | | | .06 | | | | | .0001 | | |  |
| P1NP/CTX | | | .07 | | .69 | | | | | | .10 | | | | .45 | | | | | .59 | | | .04 | | | | | .02 | | |  |
| Hormones | | |  | |  | | | | | |  | | | |  | | | | |  | | |  | | | | |  | | |  |
| PTH | | | .62 | | .75 | | | | | | .27 | | | | .79 | | | | | .34 | | | .25 | | | | | .43 | | |  |
| FGF23 | | | .001 | | .0006 | | | | | | .03 | | | | .002 | | | | | .38 | | | .05 | | | | | .35 | | |  |
| 1,25(OH)_2_D | | | .63 | | .44 | | | | | | .98 | | | | .30 | | | | | .89 | | | .80 | | | | | .29 | | |  |
| 25OHD | | | .001 | | <.0001 | | | | | | .0009 | | | | .10 | | | | | <.0001 | | | .42 | | | | | .33 | | |  |
| Plasma chemistry | | |  | |  | | | | | |  | | | |  | | | | |  | | |  | | | | |  | | |  |
| pAlb | | | .06 | | .04 | | | | | | .04 | | | | .12 | | | | | .02 | | | .12 | | | | | .33 | | |  |
| pCa | | | <.0001 | | <.0001 | | | | | | <.0001 | | | | <.0001 | | | | | <.0001 | | | .04 | | | | | .32 | | |  |
| pCa_corr_ | | | <.0001 | | <.0001 | | | | | | <.0001 | | | | <.0001 | | | | | <.0001 | | | .09 | | | | | .82 | | |  |
| pP | | | .02 | | .007 | | | | | | .91 | | | | .01 | | | | | .78 | | | .58 | | | | | .12 | | |  |
| pMg | | | .28 | | .25 | | | | | | .38 | | | | .30 | | | | | .24 | | | .75 | | | | | .41 | | |  |
| pCr | | | .34 | | .32 | | | | | | .11 | | | | .42 | | | | | .59 | | | .01 | | | | | .74 | | |  |
| Urine chemistry and renal function | | | | |  | | | | | |  | | | |  | | | | |  | | |  | | | | |  | | |  |
| eGFR | | | .35 | | .13 | | | | | | .91 | | | | .09 | | | | | .93 | | | .65 | | | | | .18 | | |  |
| TmCa/GFR | | | .67 | | .77 | | | | | | .37 | | | | .84 | | | | | .44 | | | .05 | | | | | .54 | | |  |
| TmP/GFR | | | .10 | | .06 | | | | | | .83 | | | | .05 | | | | | .50 | | | .47 | | | | | .15 | | |  |
| 2h uCa/Cr | | | .61 | | .62 | | | | | | .44 | | | | .44 | | | | | .63 | | | .11 | | | | | .83 | | |  |
| 2h uP/Cr | | | .39 | | .21 | | | | | | .44 | | | | .62 | | | | | .24 | | | .86 | | | | | .78 | | |  |
| 2h uMg/Cr | | | .03 | | .01 | | | | | | .55 | | | | .10 | | | | | .25 | | | .73 | | | | | .07 | | |  |

Data are *p*-values for the group*timepoint (tpt) interaction term from four-, three- and two-timepoint hierarchical repeated-measures ANOVA models, that included participant ID (nested by group), group, timepoint, and group*timepoint interaction. P36 = 36 weeks of pregnancy; L14, L26 = 14, 26 weeks lactation, respectively; NPNL = at least 3 months post-lactation when neither pregnant nor lactating WWH = women with HIV initiated on tenofovir-based ART during pregnancy (previously ART-naïve) who provided blood samples at 2 or more timepoints; REF = women without HIV who provided blood samples at 2 or more timepoints; ART = antiretroviral therapy; CTX = C-terminal telopeptide (ng/L); P1NP = procollagen type 1 N-terminal-propeptide (µg/L); BALP = bone-specific alkaline phosphatase (µg/L); TALP = total alkaline phosphatase U/L; P1NP/CTX = ratio of P1NP to CTX (1000*µg/ng); PTH = parathyroid hormone (ng/L); FGF23 = intact fibroblast growth factor-23 (ng/L); 1,25(OH)_2_D = 1,25-dihydroxyvitamin D (pmol/L); 25OHD = 25-hydroxyvitamin D (nmol/L); pAlb = plasma albumin (g/L); pCa = plasma calcium (mmol/L); pCa_corr_ = albumin corrected plasma calcium (Payne, mmol/L); pP = plasma phosphate (mmol/L); pMg =plasma magnesium (mmol/L); pCr= plasma creatinine (µmol/L); eGFR = estimated glomerular filtration rate (CKD, ml/min/1.73m^2^); TmCa/GFR = renal tubular maximum reabsorption of calcium per unit volume of glomerular filtrate (mmol/L); TmP/GFR = renal tubular maximum reabsorption of phosphate per unit volume of glomerular filtrate (mmol/L); 2h uCa/Cr = 2h fasting ratio of urinary calcium to creatinine 100*mmol/mmol; 2h uP/Cr = 2h fasting ratio of urinary phosphorus to creatinine (mmol/mmol); 2h uMg/Cr = 2h fasting ratio of urinary magnesium to creatinine (mmol/mmol).

^a^ The number of data points per analyte by group and timepoint are given in Supplement 3.

## Supplement 6. Characteristics and medical history by group and timepoint for participants who provided blood samples at all four timepoints.

|  | P36 | | |  | | L14 | | | |  | | L26 | | | |  | NPNL | | | | |  |  |  |
| --- | --- | --- | --- | --- | --- | --- | --- | --- | --- | --- | --- | --- | --- | --- | --- | --- | --- | --- | --- | --- | --- | --- | --- | --- |
|  | WWH (*n* = 54) | | REF (*n* = 30) |  | | WWH *(n* = 54) | | REF *(n* = 30) | |  | | WWH *(n* = 54) | | REF *(n* = 30) | |  | WWH *(n* = 54) | | | REF *(n =* 30) | |  |  |  |
|  |  | |  |  | |  | |  | |  | |  | |  | |  |  | | |  | | |  |  |
| Age (years) | | 23.5 (21.4, 27.2) | 22.7 (20.7, 26.8) | | |  | | 23.9 (21.7, 27.6) | | 23.1 (21.0, 27.2) | |  | | 24.1 (21.9, 27.8) | | 23.3 (21.3, 27.4) | | |  | 24.9 (22.9, 28.6) | | 24.3 (22.7, 28.4) | | |
| Height (cm) | | 157.0 (4.2) | 158.1 (4.5) | | |  | | 157.0 (4.2) | | 158.1 (4.5) | |  | | 157.0 (4.2) | | 158.1 (4.5) | | |  | 157.0 (4.2) | | 158.1 (4.5) | | |
| Weight (kg) | | 65.1 (59.0, 69.5) | 70.0 (65.0, 76.5) | | |  | | 56.3 (52.7, 62.7) | | 60.5 (54.3, 70.7) | |  | | 57.1 (52.4, 63.2) | | 59.7 (53.2, 69.0) | | |  | 56.8 (51.4, 63.6) | | 58.7 (53.4, 69.4) | | |
| Weeks postpartum | | - | - | | |  | | 14.3 (0.6) | | 14.1 (0.5) | |  | | 26.4 (0.6) | | 26.5 (0.6) | | |  | 67.4 (65.7, 71.3) | | 84.9 (76.6, 93.3) | | |
| BF duration (weeks) | | - | - | | |  | | 14.3 (0.6) | | 14.1 (0.5) | |  | | 26.4 (0.6) | | 26.5 (0.6) | | |  | 52.3 (51.0, 53.7) | | 71.6 (56.7, 79.9) | | |
| CD_4_ count (cells/mm^3^) | | 392 (285, 517) | - | | |  | | 396 (285, 577) | | - | |  | | 482 (331, 663) | | - | | |  | 447 (347, 680) | | - | | |
| Weeks on ART | | 11.2 (5.1) | - | | |  | | 30.0 (5.6) | | - | |  | | 42.1 (5.6) | | - | | |  | 84.2 (9.6) | | - | | |
| Pills taken %^A^ | | 99.3 (1.0) | - | | |  | | 99.7(1.4) | | - | |  | | 99.7 (1.5) | | - | | |  | 99.1 (4.3) | | - | | |
| Parity | | 1 (0, 2) | 0 (0, 1) | | |  | | 2 (1, 3) | | 1 (1, 2) | |  | | 2 (1, 3) | | 1 (1, 2) | | |  | 2 (1, 3) | | 1 (1, 2) | | |
| Nulli/primiparous % | | 37.0 | 53.3 | | |  | | 37.0 | | 53.3 | |  | | 37.0 | | 53.3 | | |  | 37.0 | | 53.3 | | |
| EBF % | | - | - | | |  | | 92.6 | | 63.3 | |  | | 87.0 | | 36.7 | | |  | - | | - | | |
| Resumed menses % | | - | - | | |  | | 40.7 | | 20.0 | |  | | 59.3 | | 43.3 | | |  | - | | - | | |
| Current DMPA % | | - | - | | |  | | 33.3 | | 13.3 | |  | | 37.0 | | 26.7 | | |  | 46.3 | | 33.3 | | |
| Prior DMPA % | | 37.0 | 16.7 | | |  | | 37.0 | | 16.7 | |  | | 37.0 | | 16.7 | | |  | 37.0 | | 16.7 | | |

Values are mean (SD) for normal distributions, median (25^th^, 75^th^ percentiles) for skewed distributions and percentage (%) for proportions of participants who reported “yes”. P36 = 36 weeks of pregnancy; L14, L26 = 14, 26 weeks lactation respectively; NPNL = at least 3 months post-lactation when neither pregnant nor lactating; WWH = women with HIV initiated on tenofovir-based ART during pregnancy (previously ART-naïve) who provided blood samples at all four timepoints; REF = women without HIV who provided blood samples at all four timepoints; ART = antiretroviral therapy; CD_4_ cell count = cells/mm^3^; EBF = exclusive breastfeeding; DMPA = depot medroxyprogesterone acetate. ^A^Mean % adherence to ART based on the pill count method used in routine clinical care = 100*[number of pills taken/number of pills dispensed for the duration]. Values of *p* for difference between groups not shown.

## Supplement 7. Biochemical markers of bone turnover and mineral metabolism by group at each timepoint; rectangular set

|  | | P36 | | | |  | | | L14 | | | |  | | | L26 | | |  | | | NPNL | | | |  |  |
| --- | --- | --- | --- | --- | --- | --- | --- | --- | --- | --- | --- | --- | --- | --- | --- | --- | --- | --- | --- | --- | --- | --- | --- | --- | --- | --- | --- |
|  | WWH (*n* = 54) | | | REF (*n* = 30) |  | | | WWH *(n* = 54) | | REF *(n* = 30) | |  | | | WWH *(n* = 54) | | REF *(n* = 30) | |  | | WWH *(n* = 54) | | | REF *(n =* 30) |  |  |  |
| Bone turnover markers | | | |  | | | |  | | | |  | | |  | | | |  | |  | | |  | | | |
| CTX ng/L | | | 511 (359, 786) | 541 (431, 681) | | | | 1106 (957, 1505) | | | | 971 (770, 1407) | | | 1012 (724, 1529) | | | | 816 (703, 1240) | | 419 (338, 617) | | | 364 (211, 561) | | | |
| P1NP µg/L | | | 55.8 (36.2, 82.6)^b^ | 74.6 (60.1, 87.0) | | | | 155 (124, 198) | | | | 155 (126, 189) | | | 141 (116, 175) | | | | 144 (120, 181) | | 110 (84, 148) | | | 90.0 (50.6, 145) | | | |
| BALP µg/L | | | 20.3 (14.6, 27.7) | 18.8 (13.3, 24.9) | | | | 40.8 (31.3, 54.7)^b^ | | | | 33.2 (25.9, 42.1) | | | 50.2 (39.3, 64.3)^a^ | | | | 37.5 (28.6, 47.7) | | 35.0 (26.9, 43.7)^a^ | | | 25.4 (16.3, 39.2) | | | |
| TALP U/L | | | 142 (115, 181) | 149 (108, 196) | | | | 141 (112, 176)^a^ | | | | 114 (97, 137) | | | 140 (116, 170)^b^ | | | | 117 (96, 147) | | 121 (105, 142)^a^ | | | 90.8 (65.4, 128) | | | |
| P1NP/CTX 1000*µg/ng | | | 109 (83, 136) | 138 (103, 158) | | | | 140 (98, 192) | | | | 160 (116, 217) | | | 139 (107, 180) | | | | 177 (135, 234) | | 262 (191, 361) | | | 248 (175, 349) | | | |
| Hormones and vitamin D status | | | |  | | | |  | | | |  | | |  | | | |  | |  | | |  | | | |
| PTH ng/L | | | 33.0 (23.7, 47.0)^a^ | 22.6 (17.2, 33.4) | | | | 62.5 (44.8, 86.2)^a^ | | | | 40.2 (28.9, 59.4) | | | 64.4 (49.3, 85.3)^a^ | | | | 42.5 (31.2, 56.1) | | 54.1 (41.4, 69.1)^b^ | | | 40.3 (28.3, 51.9) | | | |
| FGF23 ng/L | | | 4.82 (3.00, 6.82) | 4.21 (3.25, 4.53) | | | | 10.6 (7.65, 13.6) | | | | 12.7 (8.8, 13.8) | | | 10.4 (7.65, 12.3) | | | | 13.3 (9.28, 15.4) | | 9.55 (6.99, 11.2) | | | 9.42 (6.78, 12.1) | | | |
| 1,25(OH)_2_D pmol/L | | | 259 (224, 304)^b^ | 305 (261, 343) | | | | 178 (150, 207) | | | | 193 (154, 238) | | | 177 (152, 197) | | | | 191 (173, 213) | | 172 (143, 201) | | | 189 (164, 223) | | | |
| 25OHD nmol/l | | | 68.5 (58.8, 87.0) | 70.1 (55.2, 83.3) | | | | 67.4 (55.3, 95.7) | | | | 59.1 (49.9, 70.5) | | | 62.7 (51.3, 76.3)^b^ | | | | 74.8 (63.0, 88.3) | | 60.3 (52.2, 78.1) | | | 67.6 (56.9, 80.6) | | | |
| Plasma chemistry | | | |  | | | |  | | | |  | | |  | | | |  | |  | | |  | | | |
| pAlb g/L | | | 26.4 (24.6, 28.4) | 26.4 (24.9, 27.5) | | | | 37.5 (35.7, 39.6) | | | | 38.4 (36.9, 40.7) | | | 37.7 (36.0, 40.3) | | | | 37.2 (35.7, 39.6) | | 37.2 (35.9, 39.0) | | | 37.9 (36.4, 39.6) | | | |
| pCa mmol/L | | | 2.18 (2.09, 2.24) | 2.20 (2.14, 2.26) | | | | 2.22 (2.16, 2.30)^a^ | | | | 2.36 (2.30, 2.43) | | | 2.19 (2.11, 2.27) | | | | 2.17 (2.11, 2.23) | | 2.36 (2.29, 2.42) | | | 2.40 (2.35, 2.47) | | | |
| pCa_corr_ mmol/L | | | 2.44 (2.39, 2.50) | 2.47 (2.42, 2.52) | | | | 2.27 (2.18, 2.36)^a^ | | | | 2.39 (2.34, 2.45) | | | 2.24 (2.18, 2.29) | | | | 2.23 (2.18, 2.28) | | 2.41 (2.37, 2.49) | | | 2.44 (2.38, 2.50) | | | |
| pP mmol/L | | | 1.11 (0.99, 1.23)^c^ | 1.21 (1.08, 1.35) | | | | 1.29 (1.18, 1.45) | | | | 1.31 (1.24, 1.48) | | | 1.19 (1.10, 1.31) | | | | 1.23 (1.13, 1.36) | | 1.13 (1.02, 1.27) | | | 1.15 (1.03, 1.28) | | | |
| pMg mmol/L | | | 0.74 (0.70, 0.79) | 0.73 (0.70, 0.77) | | | | 0.80 (0.77, 0.83) | | | | 0.78 (0.75, 0.82) | | | 0.78 (0.74, 0.82) | | | | 0.78 (0.75, 0.82) | | 0.83 (0.79, 0.88) | | | 0.83 (0.81, 0.86) | | | |
| pCr µmol/L | | | 43.1 (38.4, 49.1) | 41.6 (37.3, 47.5) | | | | 59.7 (52.9, 67.7)^c^ | | | | 55.1 (49.8, 60.8) | | | 54.4 (47.9, 60.2)^a^ | | | | 47.9 (43.5, 53.1) | | 63.7 (57.0, 71.3) | | | 60.8 (56.8, 65.8) | | | |
| Urine chemistry and renal function | | | |  | | | |  | | | |  | | |  | | | |  | |  | | |  | | | |
| eGFR ml/min/1.73m^2^ | | | 137 (130, 143) | 139 (130, 145) | | | | 117 (109, 128)^b^ | | | | 124 (120, 131) | | | 123 (121, 131)^a^ | | | | 132 (126, 140) | | 110 (101, 123)^b^ | | | 117 (113, 127) | | | |
| TmCa/GFR mmol/L | | | 2.71 (2.29, 3.22) | 2.64 (2.36, 2.92) | | | | 3.29 (2.70, 3.99) | | | | 3.28 (2.61, 3.82) | | | 3.20 (2.86, 3.72) | | | | 2.98 (2.57, 3.72) | | 3.38 (3.13, 3.76) | | | 3.51 (3.05, 4.06) | | | |
| TmP/GFR mmol/L | | | 1.27 (1.09, 1.46)^b^ | 1.46 (1.29, 1.59) | | | | 1.48 (1.35, 1.68) | | | | 1.56 (1.38, 1.79) | | | 1.38 (1.23, 1.53) | | | | 1.52 (1.36, 1.71) | | 1.27 (1.13, 1.51) | | | 1.36 (1.18, 1.67) | | | |
| 2h uCa/Cr 100*mmol/mmol | | | 3.26 (1.61, 6.72) | 4.07 (2.28, 8.52) | | | | 1.04 (0.43, 2.34) | | | | 1.61 (0.55, 4.41) | | | 1.11 (0.55, 1.95) | | | | 1.73 (0.65, 3.25) | | 1.08 (0.66, 1.88) | | | 1.11 (0.74, 1.81) | | | |
| 2h uP/Cr mmol/mmol | | | 1.35 (1.03, 1.73) | 1.20 (0.96, 1.53) | | | | 1.14 (0.85, 1.58) | | | | 1.04 (0.87, 1.52) | | | 1.13 (0.75. 1.66) | | | | 0.88 (0.55, 1.57) | | 0.99 (0.66, 1.39) | | | 0.80 (0.46, 1.35) | | | |
| 2h uMg/Cr mmol/mmol | | | 0.18 (0.13, 0.25) | 0.21 (0.15, 0.28) | | | | 0.19 (0.14, 0.28) | | | | 0.17 (0.13, 0.26) | | | 0.23 (0.15, 0.33) | | | | 0.19 (0.15, 0.28) | | 0.21 (0.16, 0.30) | | | 0.18 (0.13, 0.25) | | | |

Data are geometric means (25^th^, 75^th^ percentiles). P36 = 36 weeks of pregnancy; L14, L26 = 14, 26 weeks lactation respectively; NPNL = at least 3 months post-lactation when neither pregnant nor lactating; WWH = women with HIV initiated on tenofovir-based ART during pregnancy (previously ART-naïve) who provided blood samples at all four timepoints (n=54); REF = women without HIV who provided blood samples at all four timepoints (n=30); ART = antiretroviral therapy; CTX = C-terminal telopeptide; P1NP = procollagen type 1 N-terminal-propeptide; BALP = bone-specific alkaline phosphatase; TALP = total alkaline phosphatase; P1NP/CTX = ratio of P1NP to CTX; PTH = parathyroid hormone; FGF23 = intact fibroblast growth factor-23; 1,25(OH)_2_D = 1,25-dihydroxyvitamin D; 25OHD = 25-hydroxyvitamin D; pAlb = plasma albumin; pCa = plasma calcium; pCa_corr_ = albumin corrected plasma calcium (Payne); pP = plasma phosphate; pMg =plasma magnesium; pCr= plasma creatinine; eGFR = estimated glomerular filtration rate (CKD); TmCa/GFR = renal tubular maximum reabsorption of calcium per unit volume of glomerular filtrate; TmP/GFR = renal tubular maximum reabsorption of phosphate per unit volume of glomerular filtrate; 2h uCa/Cr = 2h fasting ratio of urinary calcium to creatinine; 2h uP/Cr = 2h fasting ratio of urinary phosphorus to creatinine; 2h uMg/Cr = 2h fasting ratio of urinary magnesium to creatinine.

^a,b,c^ Values of *p* for difference between the groups from Scheffé post hoc tests for group*timepoint interaction terms in four-timepoint hierarchical repeated-measures ANOVA models, that included participant ID (nested by group), group, timepoint, and group*timepoint interaction. Variables were transformed into natural logarithms and multiplied by 100 before data analysis.

^a^p ≤ .001.

^b^p ≤ .01.

^c^p ≤ .05.

## Supplement 8. Within-individual changes in biochemical markers by group from pregnancy to lactation and during lactation: rectangular set.

|  | | P36 to L14 | | | |  | L14 to L26 | | | | | | | |  |
| --- | --- | --- | --- | --- | --- | --- | --- | --- | --- | --- | --- | --- | --- | --- | --- |
|  | WWH  %∆ (95% CI) | | *p* | REF  %∆ (95% CI) | *p* | | |  | | WWH  %∆ (95% CI) | *p* | REF  %∆ (95% CI) | | *p* |  |
| Bone turnover markers | | |  |  |  | | |  | |  |  |  | |  |  |
| CTX | +75.8 (+59.7, +91.9) | | <.0001 | +58.4 (+37.2, +79.6) | <.0001 | | |  | | -7.5 (-23.6, +8.6) | .84 | -17.4 (-38.6, +3.8) | | .46 |  |
| P1NP | +100.5 (87.2, +113.8) | | <.0001 | +73.2 (+55.6, +90.8) | <.0001 | | |  | | -7.9 (-21.2, +5.4) | .72 | -7.2 (-24.8, +10.4) | | .89 |  |
| BALP | +68.5 (+59.1, +77.9) | | <.0001 | +56.8 (+44.3, +69.3) | <.0001 | | |  | | +22.3 (+12.9, +31.7) | .0001 | +10.9 (-1.6, +23.4) | | .42 |  |
| TALP | -0.9 (-9.5, +7.7) | | .99 | -26.6 (-38.2, -15.0) | .0002 | | |  | | -0.3 (-8.9, +8.3) | .99 | +2.2 (-9.4, +13.8) | | .99 |  |
| P1NP/CTX | +24.7 (+8.8, +40.6) | | .03 | +14.8 (-6.2, +35.8) | .59 | | |  | | -0.4 (-16.3, +15.5) | .99 | +10.1 (-10.7, +31.1) | | .82 |  |
| Hormones and vitamin D status | | |  |  |  | | |  |  | |  | |  |  | |
| PTH | +63.8 (+50.8, +77.0) | | <.0001 | +57.6 (+40.4, +74.8) | <.0001 | | |  | | +3.1 (-10.0, +16.2) | .97 | +5.7 (-11.5, +22.9) | | .94 |  |
| FGF23 | +81.1 (+66.0, +96.2) | | <.0001 | +110.4 (+90.4, +130.4) | <.0001 | | |  | | -4.7 (-15.6, +14.6) | .95 | +4.5 (-15.5, +24.5) | | .98 |  |
| 1,25(OH)_2_D | -37.7 (-45.9, -29.5) | | <.0001 | -45.6 (-56.6, -34.6) | <.0001 | | |  | | -0.2 (-8.4, +8.0) | .99 | -1.2 (-12.2, +9.8) | | .99 |  |
| 25OHD | -1.9 (-10.3, +6.5) | | .98 | -17.1 (-28.5, -5.7) | .03 | | |  | | -6.9 (-15.3, +1.5) | .47 | +23.6 (+12.2, +35.0) | | .001 |  |
| Plasma chemistry |  | |  |  |  | | |  | |  |  |  | |  |  |
| pAlb | +35.1 (+32.6, +37.6) | | <.0001 | +37.6 (+34.1, +41.1) | <.0001 | | |  | | -0.4 (-2.9, +2.1) | .99 | -3.1 (-6.6, +0.4) | | .39 |  |
| pCa | +2.2 (+0.8, +3.6) | | .03 | +7.4 (+5.4, +9.4) | <.0001 | | |  | | -1.5 (-2.9, -0.1) | .23 | -8.4 (-10.4, -6.4) | | <.0001 |  |
| pCa_corr_ | -7.3 (-8.5, -6.1) | | <.0001 | -3.0 (-4.6, -1.4) | .006 | | |  | | -1.6 (-2.8, -0.4) | .11 | -7.2 (-8.8, -5.6) | | <.0001 |  |
| pP | +15.4 (+10.7, +20.1) | | <.0001 | +8.2 (+1.9, +14.5) | .09 | | |  | | -8.0 (-12.7, -3.3) | .01 | -6.3 (-12.6, 0.0) | | .27 |  |
| pMg | +7.6 (+5.2, +10.0) | | <.0001 | +6.7 (+3.6, +9.8) | .0005 | | |  | | -2.3 (-4.7, +0.1) | .29 | -0.3 (-3.4, +2.8) | | .99 |  |
| pCr | +32.6 (+27.9, +37.3) | | <.0001 | +28.0 (+21.7, +34.3) | <.0001 | | |  | | -9.3 (-14.0, -4.6) | .002 | -14.1 (-20.4, -7.8) | | .0003 |  |
| Urine chemistry and renal function | | |  |  |  | | |  | |  |  |  | |  |  |
| eGFR | -14.8 (-17.5, -12.1) | | <.0001 | -11.3 (-15.0, -7.6) | <.0001 | | |  | | +5.1 (+2.4, +7.8) | .005 | +6.3 (+2.6, +10.0) | | .01 |  |
| TmCa/GFR | +19.1 (+11.7, +26.5) | | <.0001 | +21.8 (+12.0, +31.6) | .0004 | | |  | | -2.9 (-10.3, +4.5) | .90 | -9.5 (-19.3, +0.3) | | .31 |  |
| TmP/GFR | +15.2 (+9.1, +21.3) | | <.0001 | +6.6 (-1.4, +14.6) | .45 | | |  | | -7.1 (-13.0, -1.2) | .15 | -3.2 (-11.2, +4.8) | | .89 |  |
| 2h uCa/Cr | -114.1 (-151.3, -76.9) | | <.0001 | -93.0 (-143.2, -43.6) | .004 | | |  | | +6.8 (-30.2, +43.8) | .99 | +7.6 (-42.2, +57.4) | | .99 |  |
| 2h uP/Cr | -16.3 (-33.9, +1.3) | | .36 | -14.3 (-37.8, +9.2) | .70 | | |  | | -2.2 (-19.9, +15.4) | .99 | -16.9 (-40.4, +6.6) | | .58 |  |
| 2h uMg/Cr | +5.9 (-11.5, +23.3) | | .93 | -17.5 (-40.6, +5.6) | .53 | | |  | | +16.9 (-0.3, +34.1) | .30 | +11.1 (-12.0, +34.2) | | .83 |  |

Values are within-individual mean percent changes (%∆) and 95% confidence intervals (95% CI) obtained from Scheffé post hoc tests for group* timepoint interaction terms in four-timepoint hierarchical repeated-measures ANOVA models, that included participant ID (nested by group), group, timepoint, and group*timepoint interaction. Variables were transformed into natural logarithms and multiplied by 100 before data analysis. The + or - signs show the direction of within-group changes (increase or decrease, respectively). P36 = 36 weeks of pregnancy; L14, L26 = 14, 26 weeks lactation respectively; WWH = women with HIV initiated on tenofovir-based ART during pregnancy (previously ART-naïve) who provided blood samples at all four timepoints; (*n* = 54); REF = women without HIV who provided blood samples at all four timepoints (*n* = 30); ART = antiretroviral therapy; CTX = C-terminal telopeptide (ng/L); P1NP = procollagen type 1 N-terminal-propeptide (µg/L); BALP = bone-specific alkaline phosphatase (µg/L); TALP = total alkaline phosphatase U/L; P1NP/CTX = ratio of P1NP to CTX (1000*µg/ng); PTH = parathyroid hormone (ng/L); FGF23 = intact fibroblast growth factor-23 (ng/L); 1,25(OH)_2_D = 1,25-dihydroxyvitamin D (pmol/L); 25OHD = 25-hydroxyvitamin D (nmol/L); pAlb = plasma albumin (g/L); pCa = plasma calcium (mmol/L); pCa_corr_ = albumin corrected plasma calcium (Payne, mmol/L); pP = plasma phosphate (mmol/L); pMg =plasma magnesium (mmol/L); pCr= plasma creatinine (µmol/L); eGFR = estimated glomerular filtration rate (CKD, ml/min/1.73m^2^); TmCa/GFR = renal tubular maximum reabsorption of calcium per unit volume of glomerular filtrate (mmol/L); TmP/GFR = renal tubular maximum reabsorption of phosphate per unit volume of glomerular filtrate (mmol/L); 2h uCa/Cr = 2h fasting ratio of urinary calcium to creatinine 100*mmol/mmol; 2h uP/Cr = 2h fasting ratio of urinary phosphorus to creatinine (mmol/mmol); 2h uMg/Cr = 2h fasting ratio of urinary magnesium to creatinine (mmol/mmol).

## Supplement 9. Within-individual changes in biochemical markers to 3 months post-lactation by group; rectangular dataset

|  | | L26 to NPNL | | | | | |  | | | |  | | P36 to NPNL | | | | | |
| --- | --- | --- | --- | --- | --- | --- | --- | --- | --- | --- | --- | --- | --- | --- | --- | --- | --- | --- | --- |
|  | WWH  %∆ (95% CI) | | *p* | REF  %∆ (95% CI) | | *p* | | |  | | | | WWH  %∆ (95% CI) | | *p* | REF  %∆ %∆ (95% CI) | *p* | |  |
| Bone turnover markers |  | |  | |  | |  | | |  |  | | | |  |  |  |  |  |
| CTX | -88.1 (-104.0, -72.2) | | <.0001 | | -80.8 (-102.0, -59.6) | | <.0001 | | |  | -19.9 (-35.8, -4.0) | | | | .11 | -39.8 (-61.0, -18.6) | .004 |  |  |
| P1NP | -24.9 (-38.0, -11.8) | | .004 | | -47.2 (-64.8, -29.6) | | <.0001 | | |  | +67.7 (+54.6, +80.8) | | | | <.0001 | +18.8 (+1.2, +36.4) | .22 |  |  |
| BALP | -34.9 (-44.3, -25.5 ) | | <.0001 | | -37.4 (-49.9, -24.9) | | <.0001 | | |  | +55.9 (+46.5, +65.3) | | | | <.0001 | +30.3 (+17.8, +42.8) | <.0001 |  |  |
| TALP | -14.8 (-23.4, -6.2) | | .01 | | -25.2 (-36.8, -13.6) | | .0005 | | |  | -16.0 (-24.6, -7.4) | | | | .005 | -49.7 (-61.3, -38.1) | <.0001 |  |  |
| P1NP/CTX | +63.3 (+47.6, +79.0) | | <.0001 | | +33.6 (+12.6, +54.6) | | .02 | | |  | +87.6 (+71.9, +103.3) | | | | <.0001 | +58.6 (+37.6, +79.6) | <.0001 |  |  |
| Hormones and vitamin D status | | |  | |  | |  | | |  |  | | | |  |  |  |  |  |
| PTH | -17.5 (-30.4, -4.6) | | .07 | | -5.4 (-22.6, +11.8) | | .95 | | |  | +49.4 (+36.5, +62.3) | | | | <.0001 | +58.0 (+40.8. +75.2) | <.0001 |  |  |
| FGF23 | -8.1 (-23.0, +6.8) | | .77 | | -34.4 (-54.4, -14.4) | | .01 | | |  | +68.4 (+53.5, +83.3) | | | | <.0001 | +80.5 (+60.5, +100.5) | <.0001 |  |  |
| 1,25(OH)_2_D | -3.4 (-11.6, +4.8) | | .88 | | -1.8 (-13.0, +9.4) | | .99 | | |  | -41.2 (-49.4, -33.0) | | | | <.0001 | -48.6 (-59.8, -37.4) | <.0001 |  |  |
| 25OHD | -3.9 (-12.3, +4.5) | | .85 | | -10.2 (-21.6, +1.2) | | .37 | | |  | -12.7 (-21.1, -4.3) | | | | .04 | -3.7 (-15.1, +7.7) | .94 |  |  |
| Plasma chemistry |  | |  | |  | |  | | |  |  | | | |  |  |  |  |  |
| pAlb | -1.3 (-3.8, +1.2) | | .79 | | +1.9 (-1.6, +5.4) | | .76 | | |  | +34.2 (+32.2, +36.2) | | | | <.0001 | +36.4 (+32.9, +39.9) | <.0001 |  |  |
| pCa | +7.3 (+5.9, +8.7) | | <.0001 | | +10.0 (+8.0, +12.0) | | <.0001 | | |  | +7.9 (+6.5, +9.3) | | | | <.0001 | +9.0 (+7.0, +11.0) | <.0001 |  |  |
| pCa_corr_ | +7.5 (+6.1, +8.9) | | <.0001 | | +9.2 (+7.6, +10.8) | | <.0001 | | |  | -1.4 (-2.6, -0.2) | | | | .17 | -1.1 (-2.7, +0.5) | .66 |  |  |
| pP | -5.5 (-10.2, -0.8) | | .14 | | -7.2 (-13.5, -0.9) | | .17 | | |  | +1.9 (-2.8, +6.6) | | | | .89 | -5.4 (-11.7, +0.9) | .41 |  |  |
| pMg | +6.1 (+3.7, +8.5) | | <.0001 | | +5.6 (+2.5, +8.7) | | .006 | | |  | +11.5 (+9.1, +13.9) | | | | <.0001 | +12.3 (+9.2, +15.4) | <.0001 |  |  |
| pCr | +15.8 (+11.1, +20.5) | | <.0001 | | +23.9 (+17.6, +30.2) | | <.0001 | | |  | +39.0 (+34.3, +43.7) | | | | <.0001 | +37.8 (+31.5, +44.1) | <.0001 |  |  |
| Urine chemistry and renal function | | |  | |  | |  | | |  |  | | | |  |  |  |  |  |
| eGFR | -11.2 (-8.5, -13.9) | | <.0001 | | -11.9 (-15.6, -8.2) | | <.0001 | | |  | -20.8 (-23.5, -18.1) | | | | <.0001 | -17.0 (-20.7, -13.3) | <.0001 |  |  |
| TmCa/GFR | +5.8 (-1.6, +13.2) | | .50 | | +16.3 (+6.5, +26.1) | | .02 | | |  | +22.0 (+14.6, +29.4) | | | | <.0001 | +28.6 (+18.8, +38.4) | <.0001 |  |  |
| TmP/GFR | -7.8 (-13.9, -1.7) | | .09 | | -10.9 (-18.9, -2.9) | | .07 | | |  | +0.4 (-5.7, +6.5) | | | | .99 | -7.5 (-15.5, +0.5) | .34 |  |  |
| 2h uCa/Cr | -2.4 (-39.6, +34.8) | | .99 | | -44.3 (-94.1, +5.5) | | .39 | | |  | -109.7 (-147.1, -72.3) | | | | <.0001 | -129.7(-179.5, -79.9) | <.0001 |  |  |
| 2h uP/Cr | -12.0 (-29.8, +5.8) | | .63 | | -8.5 (-32.0, +15.0) | | .92 | | |  | -30.5 (-48.3, -12.7) | | | | .01 | -39.7 (-63.2. -16.2) | .01 |  |  |
| 2h uMg/Cr | -5.6 (-23.0, +11.8) | | .94 | | -7.5 (-30.6, +15.6) | | .94 | | |  | +17.2 (-0.2, +34.6) | | | | .30 | -13.9 (-37.0, +9.2) | .71 |  |  |

Values are within-individual mean percent changes (%∆) and 95% confidence intervals (95% CI) obtained from Scheffé post hoc tests for group* timepoint interaction terms in four-timepoint hierarchical repeated-measures ANOVA models, that included participant ID (nested by group), group, timepoint, and group*timepoint interaction. Variables were transformed into natural logarithms and multiplied by 100 before data analysis. The + or - signs show the direction of within-group changes (increase or decrease, respectively). P36 = 36 weeks of pregnancy; L26 = 26 weeks lactation; NPNL = at least 3 months post-lactation when either pregnant nor lactation; WWH = women with HIV initiated on tenofovir-based ART during pregnancy (previously ART-naïve) who provided blood samples at all four timepoints; (*n* = 54) REF = women without HIV who provided blood samples at all four timepoints (*n* = 30) ; ART = antiretroviral therapy; CTX = C-terminal telopeptide (ng/L); P1NP = procollagen type 1 N-terminal-propeptide (µg/L); BALP = bone-specific alkaline phosphatase (µg/L); TALP = total alkaline phosphatase U/L; P1NP/CTX = ratio of P1NP to CTX (1000*µg/ng); PTH = parathyroid hormone (ng/L); FGF23 = intact fibroblast growth factor-23 (ng/L); 1,25(OH)_2_D = 1,25-dihydroxyvitamin D (pmol/L); 25OHD = 25-hydroxyvitamin D (nmol/L); pAlb = plasma albumin (g/L); pCa = plasma calcium (mmol/L); pCa_corr_ = albumin corrected plasma calcium (Payne, mmol/L); pP = plasma phosphate (mmol/L); pMg =plasma magnesium (mmol/L); pCr= plasma creatinine (µmol/L); eGFR = estimated glomerular filtration rate (CKD, ml/min/1.73m^2^); TmCa/GFR = renal tubular maximum reabsorption of calcium per unit volume of glomerular filtrate (mmol/L); TmP/GFR = renal tubular maximum reabsorption of phosphate per unit volume of glomerular filtrate (mmol/L); 2h uCa/Cr = 2h fasting ratio of urinary calcium to creatinine 100*mmol/mmol; 2h uP/Cr = 2h fasting ratio of urinary phosphorus to creatinine (mmol/mmol); 2h uMg/Cr = 2h fasting ratio of urinary magnesium to creatinine (mmol/mmol).

## Supplement 10. Summary of percentage differences between WWH and REF at each timepoint; rectangular dataset.

|  | P36 | |  | L14 | |  | L26 | |  | NPNL | |
| --- | --- | --- | --- | --- | --- | --- | --- | --- | --- | --- | --- |
|  | % Diff (95% CI) | *p* |  | % Diff (95% CI) | *p* |  | % Diff (95% CI) | *p* |  | % Diff (95% CI) | *p* |
| Bone turnover markers | |  |  |  |  |  |  |  |  |  |  |
| CTX | -5.7 (-19.3, +17.9) | .95 |  | +11.6 (+8.7, +14.5) | .69 |  | +21.6 (+3.0, +40.2) | .17 |  | +14.2 (-4.4, +32.8) | .53 |
| P1NP | -29.0 (-44.5, -13.5) | .004 |  | -1.7 (-17.4, +14.0) | .99 |  | -2.4 (-17.9, +13.1) | .99 |  | +19.9 (+4.4, +35.4) | .10 |
| BALP | +7.5 (-3.5, +18.5) | .61 |  | +19.2 (+8.0, +30.4) | .01 |  | +30.6 (+19.4, +41.8) | <.0001 |  | +33.2 (+22.0, +44.4) | <.0001 |
| TALP | -5.1 (-15.3, +5.1) | .81 |  | +20.7 (+10.5, +30.9) | .001 |  | +18.2 (+8.0, +28.4) | .007 |  | +28.6 (+18.4, +38.8) | <.0001 |
| P1NP/CTX | -23.4 (-41.8, -5.0) | .11 |  | -13.4 (-32.0, +5.2) | .58 |  | -24.0 (-42.4, -5.6) | .09 |  | +5.7 (-12.7, +24.1) | .95 |
| Hormones and vitamin D status | |  |  |  |  |  |  |  |  |  |  |
| PTH | +38.0 (+22.7, +53.3) | <.0001 |  | +44.1 (+28.6, +59.6) | <.0001 |  | +41.5 (+26.2, +56.8) | <.0001 |  | +29.4 (+14.1, +44.7) | .003 |
| FGF23 | +13.5 (-4.1, +31.1) | .52 |  | -15.7 (-33.5, +2.1) | .40 |  | -24.9 (-42.5, -7.3) | .06 |  | +1.4 (-16.2, +19.0) | .99 |
| 1,25(OH)_2_D | -16.4 (-26.0, -6.8) | .01 |  | -8.4 (-18.0, +1.2) | .41 |  | -7.3 (-16.9, +2.3) | .52 |  | -9.0 (-18.8, +0.8) | .36 |
| 25OHD | -2.4 (-12.4, +10.0) | .98 |  | +12.8 (+2.8, +22.8) | .10 |  | -17.7 (-27.7, -7.7) | .008 |  | -11.4 (-21.4, -1.4) | .18 |
| Plasma chemistry | |  |  |  |  |  |  |  |  |  |  |
| pAlb | +0.2 (-2.7, +3.1) | .99 |  | -2.2 (-5.1, +0.7) | .55 |  | +1.3 (-1.6, +4.2) | .88 |  | -2.0 (-4.9, +0.9) | .64 |
| pCa | -0.9 (-2.7, +0.9) | .80 |  | -6.1 (-7.9, -4.3) | <.0001 |  | +0.8 (-1.0, +2.6) | .86 |  | -1.9 (-3.7, -0.1) | .18 |
| pCa_corr_ | -0.9 (-2.3, +0.5) | .66 |  | -5.3 (-6.7, -3.9) | <.0001 |  | +0.4 (-1.0, +1.8) | .96 |  | -1.3 (-2.7, +0.1) | .40 |
| pP | -9.0 (-14.5, -3.5) | .02 |  | -1.8 (-7.3, +3.7) | .94 |  | -3.4 (-8.9, +2.1) | .69 |  | -1.7 (-7.2, +3.8) | .94 |
| pMg | +1.1 (-1.6, +3.8) | .89 |  | +2.1 (-0.6, +4.8) | .52 |  | -0.2 (-2.9, +2.5) | .99 |  | +0.3 (-2.4, +3.0) | .99 |
| pCr | +3.4 (-2.1, +8.9) | .68 |  | +8.1 (+2.6, +13.6) | .04 |  | +12.8 (+7.3, +18.3) | .0002 |  | +4.6 (-0.9, +10.1) | .45 |
| Urine chemistry and renal function | | |  |  |  |  |  |  |  |  |  |
| eGFR | -2.4 (-5.7, +0.9) | .55 |  | -5.9 (-9.2, -2.6) | .008 |  | -7.1 (-10.4, -3.8) | .0007 |  | -6.3 (-9.6, -3.0) | .003 |
| TmCa/GFR | +2.9 (-5.7, +11.5) | .93 |  | +0.2 (-8.4, +8.8) | .99 |  | +6.8 (-1.8, +15.4) | .50 |  | -3.7 (-12.3, +4.9) | .88 |
| TmP/GFR | -14.2 (-21.3, -7.1) | 0.002 |  | -5.7 (-12.8, +1.4) | .48 |  | -9.5 (-16.6, -2.4) | .07 |  | -6.4 (-13.5, +0.7) | .37 |
| 2h uCa/Cr | -22.8 (-66.9, +21.3) | .79 |  | -43.9 (-87.8, 0.0) | .28 |  | -44.7 (-88.6, -0.8) | .27 |  | -2.8 (-45.9, +40.3) | .99 |
| 2h uP/Cr | +11.1 (-9.7, +31.9) | .78 |  | +9.2 (-11.6, +30.0) | .86 |  | +23.8 (+3.0, +44.6) | .17 |  | +20.4 (-0.6, +41.4) | .31 |
| 2h uMg/Cr | -12.7 (-33.3, +7.9) | .69 |  | +10.7 (-9.7, +31.1) | .79 |  | +16.5 (-3.9, +36.9) | .47 |  | +18.4 (-2.2, +39.0) | .38 |

Values are mean percentage differences between the groups (%Diff) and 95% confidence intervals (95% CI) for comparison of women with HIV initiated onto TDF-based antiretroviral therapy in pregnancy (WWH) versus women without HIV (REF). Results were obtained from Scheffé post hoc tests for group* timepoint interaction terms in four-timepoint hierarchical repeated-measures ANOVA models, that included participant ID (nested by group), group, timepoint, and group*timepoint interaction. Variables were transformed into natural logarithms and multiplied by 100 before data analysis. The + or - signs show the direction of within-group changes (increase or decrease, respectively). P36 = 36 weeks of pregnancy; L14, L26 = 14, 26 weeks lactation respectively; WWH = women with HIV initiated on tenofovir-based ART during pregnancy (previously ART-naïve) who provided blood samples at all four timepoints; (*n* = 54); REF = women without HIV who provided blood samples at all four timepoints (*n* = 30); CTX = C-terminal telopeptide (ng/L); P1NP = procollagen type 1 N-terminal-propeptide (µg/L); BALP = bone-specific alkaline phosphatase (µg/L); TALP = total alkaline phosphatase U/L; P1NP/CTX = ratio of P1NP to CTX (1000*µg/ng); PTH = parathyroid hormone (ng/L); FGF23 = intact fibroblast growth factor-23 (ng/L); 1,25(OH)_2_D = 1,25-dihydroxyvitamin D (pmol/L); 25OHD = 25-hydroxyvitamin D (nmol/L); pAlb = plasma albumin (g/L); pCa = plasma calcium (mmol/L); pCa_corr_ = albumin corrected plasma calcium (Payne, mmol/L); pP = plasma phosphate (mmol/L); pMg =plasma magnesium (mmol/L); pCr= plasma creatinine (µmol/L); eGFR = estimated glomerular filtration rate (CKD, ml/min/1.73m^2^); TmCa/GFR = renal tubular maximum reabsorption of calcium per unit volume of glomerular filtrate (mmol/L); TmP/GFR = renal tubular maximum reabsorption of phosphate per unit volume of glomerular filtrate (mmol/L); 2h uCa/Cr = 2h fasting ratio of urinary calcium to creatinine 100*mmol/mmol; 2h uP/Cr = 2h fasting ratio of urinary phosphorus to creatinine (mmol/mmol); 2h uMg/Cr = 2h fasting ratio of urinary magnesium to creatinine (mmol/mmol).

**Supplement 11.** Significance of differences between WWH and REF in patterns of change between timepoints; rectangular dataset.

|  | |  | | | | |  | | |  | | | | | |  | | | |  | | | | | |  | | | | |  | | | | |  |  |  |
| --- | --- | --- | --- | --- | --- | --- | --- | --- | --- | --- | --- | --- | --- | --- | --- | --- | --- | --- | --- | --- | --- | --- | --- | --- | --- | --- | --- | --- | --- | --- | --- | --- | --- | --- | --- | --- | --- | --- |
|  | 4-timepoint  group*tpt  p-value  *P36-L14-L26-NPNL* | | | | | | | 3-timepoint  group*tpt  p-value  *P36-L14-L26* | | | 3-timepoint group*tpt  p-value  *L14-L26-NPNL* | | | | | | | | 2-timepoint  group*tpt  p-value  *P36-L14* | | | 2-timepoint group*tpt  p-value  *L14-L26* | | | | | | | 2-timepoint group*tpt  p-value  *L26-NPNL* | | | 2-timepoint group*tpt  p-value  *P36-NPNL* | | | | | | |
| Bone turnover markers | | | |  | | | | |  | | | |  | |  | | | | | |  | | | |  | | | | |  | | | | |  |  |  |  |
| CTX | | | .22 | | .07 | | | | | | | .76 | | | | | .16 | | | | | | .32 | | | | .64 | | | | | | .13 | | | |  |  |
| P1NP | | | .0003 | | .02 | | | | | | | .04 | | | | | .01 | | | | | | .91 | | | | .03 | | | | | | .0005 | | | |  |  |
| BALP | | | .005 | | .009 | | | | | | | .17 | | | | | .18 | | | | | | .08 | | | | .65 | | | | | | .006 | | | |  |  |
| TALP | | | <.0001 | | .001 | | | | | | | .17 | | | | | .003 | | | | | | .60 | | | | .08 | | | | | | .0002 | | | |  |  |
| P1NP/CTX | | | .09 | | .67 | | | | | | | .08 | | | | | .48 | | | | | | .37 | | | | .03 | | | | | | .03 | | | |  |  |
| Hormones | | |  | |  | | | | | | |  | | | | |  | | | | | |  | | | |  | | | | | |  | | | |  |  |
| PTH | | | .57 | | .86 | | | | | | | .33 | | | | | .55 | | | | | | .86 | | | | .19 | | | | | | .42 | | | |  |  |
| FGF23 | | | .01 | | .005 | | | | | | | .10 | | | | | .02 | | | | | | .39 | | | | .05 | | | | | | .40 | | | |  |  |
| 1,25(OH)_2_D | | | .56 | | .34 | | | | | | | .97 | | | | | .30 | | | | | | .86 | | | | .76 | | | | | | .35 | | | |  |  |
| 25OHD | | | .0002 | | <.0001 | | | | | | | <.0001 | | | | | .03 | | | | | | <.0001 | | | | .41 | | | | | | .26 | | | |  |  |
| Plasma chemistry | | |  | |  | | | | | | |  | | | | |  | | | | | |  | | | |  | | | | | |  | | | |  |  |
| pAlb | | | .31 | | .27 | | | | | | | .15 | | | | | .26 | | | | | | .09 | | | | .10 | | | | | | .39 | | | |  |  |
| pCa | | | <.0001 | | <.0001 | | | | | | | <.0001 | | | | | <.0001 | | | | | | <.0001 | | | | .03 | | | | | | .26 | | | |  |  |
| pCa_corr_ | | | <.0001 | | <.0001 | | | | | | | <.0001 | | | | | <.0001 | | | | | | <.0001 | | | | .08 | | | | | | .66 | | | |  |  |
| pP | | | .21 | | .13 | | | | | | | .87 | | | | | .10 | | | | | | .60 | | | | .65 | | | | | | .14 | | | |  |  |
| pMg | | | .66 | | .45 | | | | | | | .45 | | | | | .54 | | | | | | .18 | | | | .84 | | | | | | .62 | | | |  |  |
| pCr | | | .09 | | .07 | | | | | | | .08 | | | | | .23 | | | | | | .21 | | | | .01 | | | | | | .81 | | | |  |  |
| Urine chemistry and renal function | | | | | |  | | | | | | | |  | | | |  | | | | | |  | | | |  | | | | | |  | | | |  |
| eGFR | | | .22 | | .08 | | | | | | | .87 | | | | | .10 | | | | | | .57 | | | | .73 | | | | | | .18 | | | |  |  |
| TmCa/GFR | | | .40 | | .59 | | | | | | | .26 | | | | | .65 | | | | | | .32 | | | | .05 | | | | | | .26 | | | |  |  |
| TmP/GFR | | | .32 | | .20 | | | | | | | .66 | | | | | .15 | | | | | | .27 | | | | .53 | | | | | | .20 | | | |  |  |
| 2h uCa/Cr | | | .50 | | .77 | | | | | | | .32 | | | | | .59 | | | | | | .98 | | | | .11 | | | | | | .49 | | | |  |  |
| 2h uP/Cr | | | .72 | | .57 | | | | | | | .60 | | | | | .91 | | | | | | .39 | | | | .84 | | | | | | .63 | | | |  |  |
| 2h uMg/Cr | | | .13 | | .08 | | | | | | | .87 | | | | | .13 | | | | | | .64 | | | | .92 | | | | | | .05 | | | |  |  |

Data are *p*-values for the group*timepoint (tpt) interaction term from four-, three- and two-timepoint hierarchical repeated-measures ANOVA models, that included participant ID (nested by group), group, timepoint, and group*timepoint interaction. P36 = 36 weeks of pregnancy; L14, L26 = 14, 26 weeks lactation respectively; NPNL = at least 3 months post-lactation when neither pregnant nor lactating WWH = women with HIV initiated on tenofovir-based ART during pregnancy (previously ART-naïve) who provided blood samples at all four timepoints (*n* = 54); REF = women without HIV who provided blood samples at at all four timepoints (*n* = 30); ART = antiretroviral therapy; CTX = C-terminal telopeptide (ng/L); P1NP = procollagen type 1 N-terminal-propeptide (µg/L); BALP = bone-specific alkaline phosphatase (µg/L); TALP = total alkaline phosphatase U/L; P1NP/CTX = ratio of P1NP to CTX (1000*µg/ng); PTH = parathyroid hormone (ng/L); FGF23 = intact fibroblast growth factor-23 (ng/L); 1,25(OH)_2_D = 1,25-dihydroxyvitamin D (pmol/L); 25OHD = 25-hydroxyvitamin D (nmol/L); pAlb = plasma albumin (g/L); pCa = plasma calcium (mmol/L); pCa_corr_ = albumin corrected plasma calcium (Payne, mmol/L); pP = plasma phosphate (mmol/L); pMg =plasma magnesium (mmol/L); pCr= plasma creatinine (µmol/L); eGFR = estimated glomerular filtration rate (CKD, ml/min/1.73m^2^); TmCa/GFR = renal tubular maximum reabsorption of calcium per unit volume of glomerular filtrate (mmol/L); TmP/GFR = renal tubular maximum reabsorption of phosphate per unit volume of glomerular filtrate (mmol/L); 2h uCa/Cr = 2h fasting ratio of urinary calcium to creatinine 100*mmol/mmol; 2h uP/Cr = 2h fasting ratio of urinary phosphorus to creatinine (mmol/mmol); 2h uMg/Cr = 2h fasting ratio of urinary magnesium to creatinine (mmol/mmol).
